# Pharmacokinetics and tissue distribution of four major bioactive components of *Cynanchum auriculatum* extract: a UPLC–MS/MS study in normal and functional dyspepsia rats

**DOI:** 10.3389/fphar.2023.1279971

**Published:** 2023-10-17

**Authors:** Jia Sun, Xin Meng, Di Huang, Zipeng Gong, Chunhua Liu, Ting Liu, Jie Pan, Yuan Lu, Lin Zheng

**Affiliations:** ^1^ State Key Laboratory of Functions and Applications of Medicinal Plants, Guizhou Provincial Key Laboratory of Pharmaceutics, Guizhou Medical University, Guiyang, China; ^2^ National Engineering Research Center of Miao’s Medicines, Guiyang, China; ^3^ School of Pharmacy, Guizhou Medical University, Guiyang, China; ^4^ Engineering Research Center for the Development and Application of Ethnic Medicine and TCM (Ministry of Education), Guiyang, China

**Keywords:** *Cynanchum auriculatum*, UPLC-MS/MS, pharmacokinetics, tissue distribution, functional dyspepsia

## Abstract

**Introduction:** *Cynanchum auriculatum* (CA) is usually used to treat digestive disorders, such as anorexia, enteritis, dysentery, and indigestion. Functional dyspepsia (FD) is characterized by a group of symptoms associated with the gastroduodenal region. Recent pharmacological studies have demonstrated the efficacy of CA for treating FD. However, the pharmacokinetics (PK) and tissue distribution of CA in physiological and FD states is still unclear. The present study aimed to clarify the differences in PK parameters and tissue distribution of the four major active components of CA (baishouwu benzophenone, deacylmet-aplexigenin, qingyangshengenin, and syringic acid) under both physiological and FD states.

**Methods:** For this, normal and FD rats were orally administered 10 mg/kg CA extract. Then, plasma and tissue (heart, liver, spleen, lung, kidney, brain, stomach, and small intestine) samples were obtained. The four active components of CA in rat plasma and tissues were quantified by developing and validating a fast and reliable ultra–high–performance liquid chromatography–mass spectrometry method.

**Results:** The area under the plasma concentration–time curve from time zero to time t (AUC_0-t_) of baishouwu benzophenone was significantly lower in the FD group than in the normal group (*p* < 0.01). The FD group had significantly lower (*p* < 0.001) apparent volume of distribution and plasma clearance of qing-yangshengenin and significantly higher (*p* < 0.05) AUC_0-t_ of deacylmetaplexigenin and qingyangshengenin. The four active components were rapidly distributed into various tissues, and the main target organs of CA activity were the stomach and small intestine. In addition, baishouwu benzophenone, deacylmetaplexigenin, and qingyangshengenin could cross the blood-brain barrier, indicating that the brain may be another target organ in the treatment of FD.

**Discussion:** These results indicate that the pathological state of FD alters the PK behavior and tissue distribution characteristics of baishouwu benzophenone, deacylmetaplexigenin, qingyangshengenin, and syringic acid in the CA extract, providing an experimental basis for the role of CA in FD treatment.

## Graphical Abstract

**Figure:**
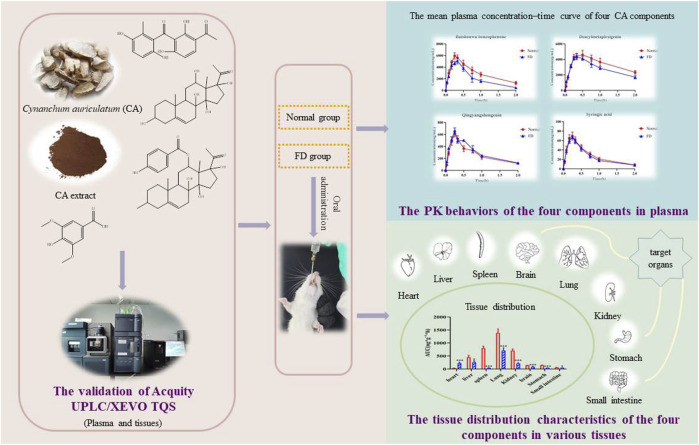

## 1 Introduction

Functional dyspepsia (FD) is a gastroduodenal disorder with an average global prevalence of 7.2%. FD remains a less well understood and heterogenous disorder. There is a significant overlap of symptoms between FD and gastroparesis and transition in diagnosis over time. (Hiroki and Madhusudan, 2023). Patients with FD often complain about abdominal pain and discomfort that mostly occurs after meals (Zheng et al., 2022). In traditional Chinese medicine (TCM), FD is accompanied by clinical symptoms such as epigastric pain, stomach distention, and a feeling of fullness (Zhang et al., 2019). Therefore, medicines that primarily promote digestion and protect the stomach are used for the treatment of FD.

The roots of *Cynanchum auriculatum* (CA) Royle ex Wight are commonly used by the ethnic minorities in Guizhou province for their medicinal properties to treat digestive disorders, such as anorexia, enteritis, dysentery, and indigestion. In addition, modern pharmacological studies have revealed the efficacy of CA for the treatment of gastric disorders such as FD, chronic atrophic gastritis, and irritable bowel syndrome (Administration, 2003; Medica, 2005). The CA preparations used by the Guizhou ethnic medicines that have been included in the national drug compendiums include Geshanxiaoji granules, Weikean capsules, Heweixiaopi capsules, and Children Xiaoshi appetizer granules. To sum up, CA have strong regional characteristics and development potential.

**FIGURE 1:**
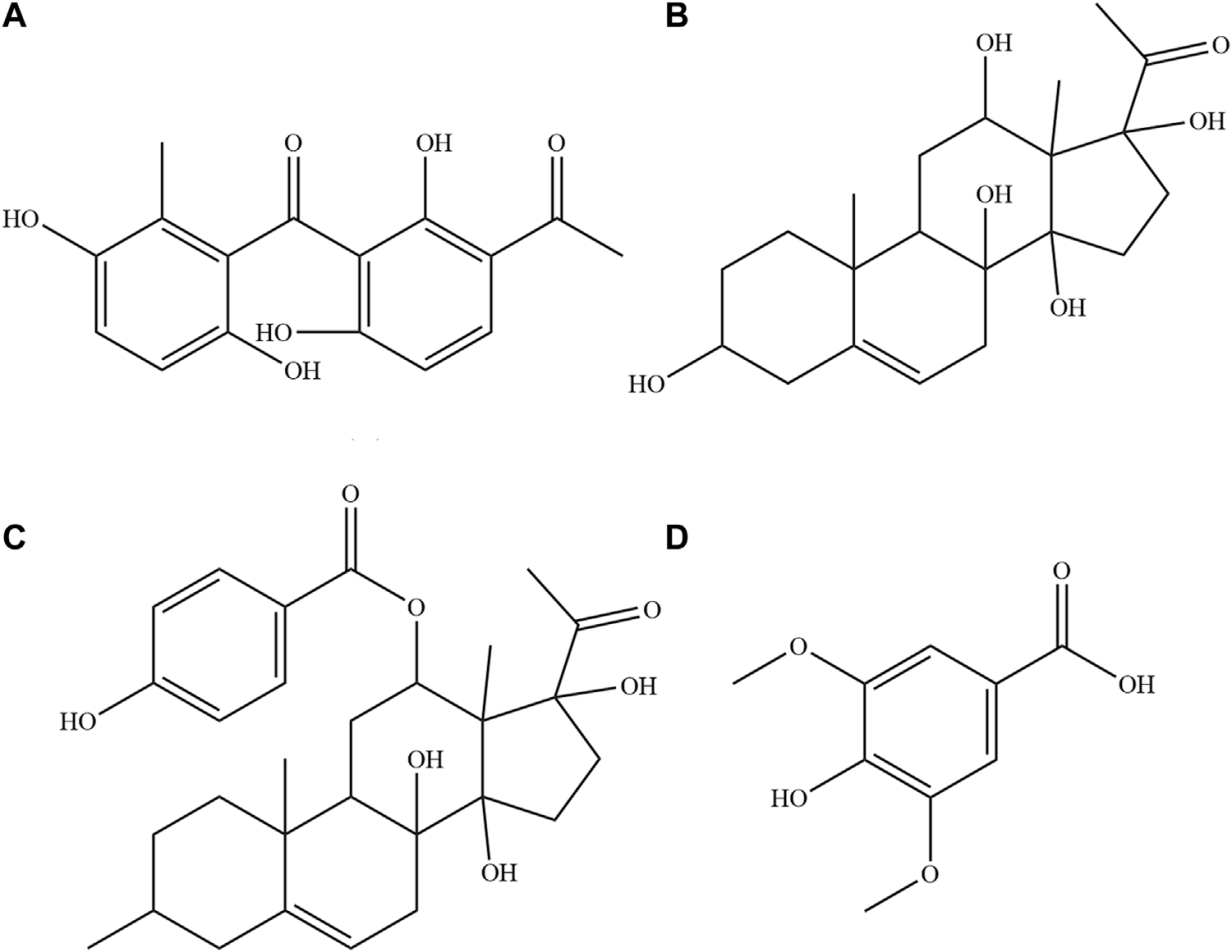
The structural formula of the four compositions in plasma and tissue homogenate samples. **(A)** Baishouwu benzophenone **(B)** Deacylmetaplexigenin **(C)** Qingyangshengenin **(D)** Syringic acid.

It is well known that for medications to exert their pharmacological effects, it is essential that they reach the target site. In addition, the concentration and residence time of the drug at the target site also determine drug efficacy. Reportedly, the drug concentration at the site of action is not only related to the dosage but also closely associated with the drug’s metabolic kinetics (Yang et al., 2022). When administered, a drug undergoes absorption, distribution, metabolism, and excretion *in vivo*, which are influenced by various factors. Physiological dysfunction caused by different diseases is an important factor affecting a drug’s kinetics, highlighting the need to study the pharmacokinetics (PK) of drugs in pathological conditions. Several studies have reported that the disease state can significantly alter the PK parameters of drugs, such as lansoprazole (Han et al., 2011), Zhishi San (Chen et al., 2014), Aidijin Injection (Liu et al., 2016), and Wuhexing Pills (Zhang et al., 2021), which are used in the treatment of gastrointestinal disorders. CA is one such medicinal agent that is used for the treatment of FD. Previous laboratory studies have found that the 60% ethanol–eluted fraction from the CA extract is the main active fraction promoting gastrointestinal motility (Liu et al., 2018). The possible anti–FD components of CA were screened, such as syringic acid, deacylmetaplexigenin, ferulic acid, scopoletin, baishouwu benzophenone, and qingyangshengenin by combining spectrum–effect relationship modeling (Sun et al., 2023). Oral absorption studies of CA extract revealed that the overall absorption of mainly anti-FD components are lower in FD state than in the normal state (Liu et al., 2022; Sun et al., 2022). It is well known that diseases induce damage to body tissues and organs, which causes abnormal regulation of various metabolic enzymes and transporters involved in drug metabolism, resulting in altered PK and pharmacodynamics of the drug (Amandla et al., 2016). After drug absorption into the blood, it needs to be distributed throughout the circulatory system to reach the respective target cells, target organs, and target tissues to exert its pharmacological effects. The tissue distribution study can uncover the distribution patterns of drugs in the body and their potential targeting effects, which is an essential aspect of guiding the clinical application of drugs. Furthermore, tissue distribution plays a significant role in toxicological studies of drugs, the assessment of long-term drug toxicity, and the safety evaluation of new drugs (Wu et al., 2016; Peng et al., 2018; Wang et al., 2021). However, the PK and tissue distribution of the four major active components of CA extract in physiological and FD states is still unclear. Therefore, the present study assessed the syringic acid, qingyangshengenin, deacylmetaplexigenin, and baishouwu benzophenone (Figure 1) as the representative active components of CA to determine the dynamic processes of these components in normal and FD rats. The findings would provide a theoretical basis for further development and utilization of CA medicinal materials.

## 2 Materials and methods

### 2.1 Materials, chemicals, and reagents

CA was collected from Kaili City, Guizhou Province of China, and identified by Prof. Chunhua Liu of the Department of Pharmacognosy, School of Pharmacy, Guizhou Medical University, China. Syringic acid (purity ≥98%) was supplied by Chengdu Push Bio–Technology Co., Ltd. (Sichuan, China). Qingyangshengenin (purity ≥98%) and puerarin (purity ≥98%) were purchased from Chengdu Alfa Biotechnology Co., Ltd. (Sichuan, China). Deacylmetaplexigenin (purity ≥99.66%) was supplied by Beijing Century Aoke Biotechnology Co., Ltd. (Beijing, China). Baishouwu benzophenone was synthesized at the School of Pharmacy, Guizhou Medical University (purity ≥98%). Acetonitrile, formic acid, and methanol (HPLC grade) were imported from Merck KGaA Technology Company (Darmstadt, Germany). N–butyl alcohol was purchased from Chongqing Wansheng East Sichuan Chemical Industry Co., Ltd. (Chongqing, China). Ethyl acetate was procured from Shanghai Shenbo Chemical Industry Co., Ltd. (Shanghai, China). All other reagents were of analytical grade.

### 2.2 Analytical conditions

UPLC–MS/MS analysis was conducted using the ACQUITY UPLC system (Waters Corp., Milford, MA, United States) equipped with a Xevo–TQ mass spectrometer (Waters Corp., Milford, MA, United States) with an electrospray ionization (ESI) interface and triple quadrupole mass analyzer.

The ACQUITY UPLC^®^ BEH C_18_ column (2.1 mm × 50 mm, 1.7 μm, Waters, United States) was used for separation and was maintained at 40°C. The mobile phase consisted of 0.1% formic acid in water (A) and 0.1% formic acid–acetonitrile (B) for PK study and 0.2% formic acid in water (A) and 0.2% formic acid–acetonitrile (B) for tissue distribution study. The mobile phase was eluted as a gradient, as follows: 0–0.5 min, 10% B; 0.5–3.0 min, 15% B; 3.0–3.2 min, 25% B; 3.2–4.0 min, 30% B; 4.0–4.5 min, 32% B; 4.5–6.0 min, 90% B; and 6.0 min, 10% B. The flow rate was set at 0.35 mL/min and the injection volume was 2 μL for the PK study and 1 μL for the tissue distribution study.

The four active components of CA were quantified using multiple reaction monitoring under negative ion mode. The following m/z transitions were chosen for quantification: baishouwu benzophenone 301.00→283.00, deacylmetaplexigenin 379.15→361.20, qingyangshengenin 499.20→137.00, syringic acid 197.20→181.95, and puerarin (internal standard, IS) 415.10→267.05. The collision energy was 15, 15, 35, 15, and 35 V, respectively (Sun et al., 2022). ESI mode was operated under the following conditions: capillary voltage, 1 kV; desolvation gas (N_2_), 1000 L/h; collision gas (Ar_2_), 150 L/h; desolvation temperature, 400°C; and source temperature, 120°C.

### 2.3 Animals and establishment of FD model

Specific pathogen–free, male Sprague–Dawley rats (230 ± 10 g) obtained from Changsha Tianqin Biotechnology (Certificate: SCXK (Xiang) 2019–0014) were housed at a controlled temperature (20°C–25°C) and humidity (40%–70%). All animals were fed with a standard rodent chow and water *ad libitum* for a week before the experiment. All rat experiments were performed and approved by the Animal Ethics Committee of Guizhou Medical University (No: 1702082).

Based on our previous research, FD was induced in rats using the modified method of ice-diluted hydrochloric acid treatment (Qin et al., 2021a). Briefly, rats were fed irregularly (alternate day fasting) and were orally administered 0.5 mol/L iced hydrochloric acid (0.8 mL/100 g/d) for 21 days to induce FD.

### 2.4 Preparation of CA extract

The CA extract was prepared as described previously (Liu et al., 2018). Briefly, the pulverized root tuber of CA was weighed and extracted thrice under reflux with 70% ethanol. The ethanol extracts obtained were combined, concentrated, and then purified by passing through D–101 macroporous resin and washing thrice with water and then with 60% ethanol. The contents of baishouwu benzophenone, deacylmetaplexigenin, qingyangshengenin, and syringic acid in the CA extract were 0.49%, 1.37%, 0.97%, and 0.01%, respectively.

### 2.5 Sample preparation

#### 2.5.1 Preparation of plasma samples

In a 1.5 mL EP tube, 100 μL of plasma sample was mixed with 10 µL 1% formic acid–water, 10 μL IS (0.2011 μg/mL), and 400 μL acetonitrile. The mixture was then vortexed for 5 min, followed by sonication for 10 min at 40 kHz and centrifugation at 14,000 rpm for 10 min. The supernatant obtained was transferred to another tube and evaporated to dryness under a nitrogen vacuum at 37°C. The residue obtained was reconstituted with 150 μL of the initial mobile phase, and the centrifugation process was repeated. The supernatant thus obtained was then used for further analysis.

#### 2.5.2 Preparation of tissue samples

All tissues were weighed and homogenized with normal saline (stomach and small intestine, v:w = 4:1; other tissues, v:w = 2:1). It is worth noting that the stomach and intestinal cavity should be repeatedly rinsed with normal saline to ensure the absence of any drug residue. Additionally, stomach and small intestinal tissues were diluted three times to obtain a tissue homogenate. Then, 300 μL of this tissue homogenate was mixed with 30 μL 1% formic acid-water, 20 μL IS, and 1.2 mL acetonitrile. The mixture was then vortexed for 5 min, sonicated for 10 min, and then stored at −20°C for 30 min. Subsequently, the mixture was centrifuged at 14,000 rpm for 10 min. The supernatant obtained was then transferred to another tube and evaporated to dryness at 37°C under nitrogen. The dried residue was reconstituted with 200 μL of the 50% methanol–water, and the centrifugation process was repeated. The obtained supernatant was then used for further analysis.

### 2.6 Preparation of calibration and quality control (QC) sample

The appropriate amount of the reference substance was accurately weighed and dissolved in an appropriate amount of methanol to obtain stock solutions. The concentrations of the resulting stock solution were 0.9996 mg/mL for baishouwu benzophenone, 0.9518 mg/mL for deacylmetaplexigenin, 1.3660 mg/mL for qingyangshengenin, and 0.9898 mg/mL for syringic acid. Accurate volumes of each stock solution were mixed to obtain working solution, which was then serially diluted with methanol to provide standard working solutions. Similarly, QC samples of three concentrations (low, medium, and high) were prepared. All solutions were stored at −20°C for later use.

### 2.7 Method validation

The established UPLC–MS/MS method for the absolute quantification of baishouwu benzophenone, qingyangshengenin, syringic acid, and deacylmetaplexigenin concentrations was fully validated in rat plasma and tissue homogenate, according to the Guidance for Industry for Bioanalytical Method Validation issued by both the United States FDA and the NMPA of China.

The specificity of the method was evaluated by comparing the retention times of analytes and IS in the blank matrix, blank matrix spiked with the four analytes and IS, and the samples after oral administration of CA extract. Calibration curves were constructed by comparing the ratio of peak areas of the four analytes to that of IS *versus* the concentration of the four analytes. Calibration curves were fitted via linear weighted (*1/X* ^*2*^) least–squares regression. The intra–and inter–day precision and accuracy were estimated by analyzing QC samples at different concentrations on the same day and three consecutive validation days, respectively. The extraction recovery was evaluated by determining the peak area of analytes in QC samples compared with that in extracted matrix samples. The relative standard deviation of the extraction recovery of analytes at each level was retained at ≤20.0%. The following parameters of stability were assessed: short–term stability (24 h at room temperature), processed sample stability (24 h at 4°C), freeze–thaw stability (3 cycles), and auto–sampler stability (6 h at room temperature). QC samples of different concentrations were used for stability analysis.

### 2.8 PK study

For PK experiments, an appropriate amount of the CA extract was weighed to prepare a 0.10 g/mL solution using physiological saline. Briefly, 12 SD rats were randomly assigned to normal and FD groups and were orally administered the prepared CA sample (10 mL/kg body weight). For assessment, 0.3 mL of blood was collected into heparinized tubes via the caudal vein before dosing and at 2, 5, 10, 15, 20, 30, and 45 min, and 1, 2, 4, 8, 12, 24, and 48 h after administration. Blood samples were then immediately centrifuged at 4,500 rpm for 15 min, and the obtained plasma samples were stored at −20°C until determination.

### 2.9 Tissue distribution study

SD rats were randomly divided into normal and FD groups (*n* = 30 each) and orally administered 10 mL/kg of CA extract solution. The rats were sacrificed at 10, 30 min, 1, 2, and 4 h after administration (6 rats per time point). Subsequently, their heart, liver, spleen, lung, kidney, brain, small intestine, and stomach were removed. The tissue samples were then washed in saline solution and wiped with filter paper. Subsequently, all tissues were stored at −80°C until analysis.

### 2.10 Data analysis

The PK parameters were analyzed using the non–compartment analysis using the WinNonLin 8.2 software (Phoenix, United States). All the data were expressed as mean ± standard deviation (SD). The normal and FD groups were compared using independent samples *t*–test in SPSS 22.0. Results with *p* values < 0.05 were considered statistically significant.

## 3 Results

### 3.1 Method validation

No endogenous interference of the four analytes and the IS was observed in the blank plasma matrix (Supplementary Figure S1). The calibration curves (Table 1) for the four compounds in plasma and tissues demonstrated good linearity over 0.0002–6.9400 μg/mL. For all the linearity ranges tested, the correlation coefficients were ≥0.9900. The results of precision, accuracy, extraction recovery, and matrix effect have been summarized in Table 2. The accuracy was from 86.06% to 115.03% in rat plasma and tissue homogenates. In addition, the intra–and inter–day precision was <15%, which met the acceptance criteria. In addition, the results of stability (Tables 3, 4) met the acceptance criteria.

**TABLE 1:** Calibration curve of the four compounds in plasma and tissue homogenate samples.

Matrix	Compound	Range (ng·mL^-1^)	Calibration curve	*R* ^2^	LOQ (ng·mL^-1^)	LOD (ng·mL^-1^)
Plasma	Baishouwu benzophenone	26.03–6664.00	*Y* = 0.0700*X*+3.1543	0.9950	0.81	0.41
	Deacylmetaplexigenin	12.39–6940.00	*Y =* 0.0175*X+*0.4817	0.9994	0.75	0.42
	Qingyangshengenin	2.22–1138.33	*Y* = 0.0108*X*+0.0022	0.9991	0.93	0.46
	Syringic acid	1.61–412.42	*Y* = 0.0034*X*+0.0072	0.9997	1.61	0.81
Heart	Baishouwu benzophenone	0.65–167.33	*Y* = 0.0607*X* −0.0286	0.9957	0.16	0.08
	Deacylmetaplexigenin	8.92–2284.32	*Y* = 0.0163*X*+0.2664	0.9986	0.56	0.28
	Qingyangshengenin	2.06–526.14	*Y* = 0.0211*X* +0.1660	0.9952	0.51	0.26
	Syringic acid	2.00–511.40	*Y* = 0.0036*X*+0.0004	0.9997	2.00	1.00
Liver	Baishouwu benzophenone	0.41–418.33	*Y* = 0.0284*X* +0.0641	0.9996	0.20	0.10
	Deacylmetaplexigenin	8.06–4124.47	*Y* = 0.0182*X*+0.9023	0.9932	0.50	0.25
	Qingyangshengenin	11.68–5978.83	*Y* = 0.0070*X* +0.7120	0.9906	0.73	0.36
	Syringic acid	6.44–412.42	*Y =* 0.0015*X*+0.0032	0.9999	6.44	3.22
Spleen	Baishouwu benzophenone	3.43–878.50	*Y* = 0.0469*X* −0.0638	0.9998	0.21	0.11
	Deacylmetaplexigenin	10.31–2639.66	*Y* = 0.0133*X*+0.0550	0.9997	0.64	0.32
	Qingyangshengenin	2.18–558.02	*Y* = 0.0274*X* −0.0023	0.9998	0.27	0.13
	Syringic acid	3.92–1003.55	*Y =* 0.0025*X-*0.0152	0.9997	3.92	1.96
lung	Baishouwu benzophenone	6.54–836.67	*Y* = 0.0446*X* +0.0278	0.9998	0.20	0.10
	Deacylmetaplexigenin	15.70–2009.36	*Y* = 0.0161*X*+0.0811	0.9991	0.49	0.25
	Qingyangshengenin	4.00–512.21	*Y* = 0.0204*X* +0.0546	0.9980	0.50	0.25
	Syringic acid	6.87–879.82	*Y =* 0.0028*X*-0.0087	0.9997	3.44	1.72
kidney	Baishouwu benzophenone	3.92–1004.00	*Y* = 0.0578*X* +0.3690	0.9995	0.25	0.12
	Deacylmetaplexigenin	11.77–3014.03	*Y* = 0.0160*X*+0.5229	0.9981	0.74	0.37
	Qingyangshengenin	4.31–1103.22	*Y* = 0.0121*X* +0.1530	0.9981	0.54	0.27
	Syringic acid	6.44–1649.67	*Y =* 0.0025*X+*0.0009	0.9999	3.22	1.61
brain	Baishouwu benzophenone	1.36–174.03	*Y* = 0.1090*X* +0.0606	0.9993	0.08	0.04
	Deacylmetaplexigenin	7.02–898.92	*Y* = 0.0210*X*+0.0453	0.9986	0.88	0.44
	Qingyangshengenin	0.64–82.47	*Y* = 0.0407*X* +0.0185	0.9985	0.32	0.16
	Syringic acid	3.33–426.16	*Y =* 0.0031*X+*0.0019	0.9999	3.33	1.66
Small intestine	Baishouwu benzophenone	0.20–52.29	*Y* = 0.1333*X* −0.0430	0.9994	0.05	0.03
	Deacylmetaplexigenin	6.28–1608.54	*Y* = 0.0127*X*+0.3227	0.9968	0.79	0.39
	Qingyangshengenin	8.99–2301.85	*Y* = 0.0056*X* +0.3017	0.9939	0.56	0.28
	Syringic acid	4.03–1031.04	*Y* = 0.0022*X*+0.0304	0.9956	4.03	2.01
stomach	Baishouwu benzophenone	0.25–62.75	*Y* = 0.1302*X* +0.0621	0.9999	0.06	0.03
	Deacylmetaplexigenin	8.68–2220.87	*Y* = 0.0129*X*+0.2103	0.9997	0.54	0.27
	Qingyangshengenin	6.70–1713.93	*Y* = 0.0086*X* +0.1180	0.9999	0.84	0.42
	Syringic acid	1.34–343.68	*Y* = 0.0074*X*+0.0120	0.9998	1.34	0.67

**TABLE 2:** The accuracies, precisions, extraction recoveries, matrix effects of the four components in plasma and tissue homogenate samples ( $\bar{x}$ ±s, *n* = 5).

Matrix	Compound	QC (ng·mL^-1^)	Mean ± SD (ng·mL^-1^)	Accuracy (%)	Precision (RSD, %)		Extraction recovery (Mean ± SD, %)	Matrix effct (Mean ± SD, %)
					Intri-	Inter-		
Plasma	Baishouwu benzophenone	52.06	55.18 ± 2.65	105.99 ± 5.10	4.81	7.42	103.79 ± 11.03	109.90 ± 5.41
		208.25	221.24 ± 11.27	106.24 ± 5.41	5.10	6.16	109.55 ± 6.98	110.77 ± 8.87
		1666.00	1570.44 ± 89.41	94.26 ± 5.37	5.69	7.04	96.12 ± 9.40	98.14 ± 14.19
	Deacylmetaplexigenin	49.57	48.25 ± 1.96	97.34 ± 3.95	4.06	5.25	100.51 ± 5.19	110.83 ± 8.04
		396.57	352.81 ± 12.43	88.97 ± 3.13	3.52	2.65	99.22 ± 5.63	95.77 ± 2.20
		3172.53	2975.48 ± 83.43	93.79 ± 2.63	2.80	2.45	94.53 ± 13.27	99.59 ± 12.79
	Qingyangshengenin	8.89	9.06 ± 0.92	101.89 ± 10.39	10.20	12.43	96.15 ± 16.07	112.25 ± 8.46
		71.13	62.14 ± 1.84	87.37 ± 2.59	2.97	3.08	96.87 ± 3.47	111.26 ± 4.37
		569.17	522.14 ± 17.90	91.74 ± 3.15	3.43	3.16	87.05 ± 13.72	102.67 ± 13.87
	Syringic acid	1.61	1.45 ± 0.10	89.95 ± 6.13	6.81	8.78	103.95 ± 3.44	104.67 ± 11.41
		25.78	23.57 ± 2.09	91.45 ± 8.10	8.86	7.04	93.74 ± 8.05	104.20 ± 12.00
		206.21	184.69 ± 4.59	89.56 ± 2.23	2.48	2.25	103.14 ± 3.39	102.27 ± 2.97
Heart	Baishouwu benzophenone	2.63	2.87 ± 0.23	108.94 ± 8.65	7.94	9.23	96.84 ± 9.87	105.37 ± 5.77
		18.80	20.72 ± 0.56	110.19 ± 2.98	2.71	3.20	99.36 ± 8.04	97.86 ± 11.27
		110.61	119.59 ± 1.62	108.12 ± 1.46	1.35	1.33	97.21 ± 3.69	95.67 ± 6.73
	Syringic acid	8.11	9.33 ± 0.24	115.03 ± 2.95	2.57	6.34	97.49 ± 8.39	101.37 ± 8.61
		57.96	55.01 ± 2.29	94.92 ± 3.95	4.16	4.71	96.72 ± 6.79	103.58 ± 9.19
		340.93	327.79 ± 5.10	95.15 ± 1.50	1.55	4.53	93.80 ± 7.73	99.06 ± 13.88
	Qingyangshengenin	8.34	8.79 ± 0.31	105.50 ± 3.67	3.48	4.98	94.68 ± 3.92	101.84 ± 7.55
		59.54	65.32 ± 2.68	109.71 ± 4.50	4.10	1.81	95.27 ± 4.31	102.59 ± 9.49
		350.22	394.79 ± 7.13	112.73 ± 2.04	1.81	0.77	92.60 ± 6.57	101.23 ± 11.21
	Deacylmetaplexigenin	36.65	35.31 ± 1.26	96.36 ± 3.44	3.57	9.25	96.47 ± 3.61	101.24 ± 6.87
		261.75	280.17 ± 3.20	107.04 ± 1.22	1.14	5.60	96.25 ± 5.11	99.36 ± 8.84
		1539.72	1676.44 ± 21.78	108.88 ± 1.41	1.30	3.69	92.94 ± 6.54	100.60 ± 11.95
Liver	Baishouwu benzophenone	1.65	1.72 ± 0.15	106.69 ± 9.08	8.67	4.42	109.95 ± 4.55	97.98 ± 9.06
		26.13	27.76 ± 2.55	106.22 ± 9.75	9.18	3.64	105.53 ± 2.14	98.91 ± 2.73
		311.09	303.98 ± 31.09	97.71 ± 9.99	10.22	2.00	104.61 ± 4.38	100.61 ± 1.04
	Syringic acid	22.34	22.89 ± 2.18	102.48 ± 9.77	9.53	6.17	112.20 ± 6.64	98.75 ± 7.23
		89.36	87.10 ± 5.52	97.47 ± 6.18	6.34	2.33	113.05 ± 5.39	97.20 ± 3.84
		268.07	294.16 ± 18.26	109.73 ± 6.78	6.21	4.65	110.98 ± 2.96	96.49 ± 1.86
	Qingyangshengenin	23.48	21.84 ± 1.35	93.05 ± 5.74	6.17	4.84	101.40 ± 1.58	94.23 ± 2.52
		372.70	371.06 ± 7.63	99.56 ± 2.05	2.06	1.47	96.93 ± 2.42	97.40 ± 1.64
		4436.88	4727.76 ± 49.58	108.81 ± 1.12	1.03	0.84	99.02 ± 2.87	97.69 ± 1.29
	Deacylmetaplexigenin	16.37	16.32 ± 0.73	99.73 ± 4.46	4.47	3.73	104.14 ± 1.99	97.97 ± 2.75
		259.83	251.40 ± 3.85	96.76 ± 1.48	1.53	3.42	101.73 ± 1.89	97.46 ± 4.73
		3093.19	2439.85 ± 105.56	109.91 ± 2.07	3.07	2.16	102.06 ± 2.39	98.14 ± 3.54
Spleen	Baishouwu benzophenone	10.89	11.30 ± 0.60	103.81 ± 5.54	5.34	0.89	102.80 ± 3.23	101.57 ± 5.84
		87.11	79.81 ± 3.51	91.62 ± 4.03	4.40	0.53	101.30 ± 7.07	98.94 ± 3.21
		653.29	685.24 ± 36.21	104.89 ± 5.54	5.28	0.53	98.25 ± 6.64	98.38 ± 8.43
	Syringic acid	12.54	11.63 ± 1.22	92.70 ± 9.75	10.51	4.54	101.73 ± 6.38	95.66 ± 2.63
		100.35	95.64 ± 3.19	95.30 ± 3.18	3.33	3.75	102.43 ± 5.87	105.23 ± 1.04
		752.66	694.38 ± 27.16	92.26 ± 3.61	3.91	1.72	100.98 ± 4.73	95.20 ± 8.08
	Qingyangshengenin	6.90	6.57 ± 0.34	95.20 ± 5.02	5.27	2.54	100.61 ± 4.43	99.45 ± 1.82
		55.21	51.78 ± 2.62	93.79 ± 4.75	5.07	1.10	98.54 ± 3.14	99.18 ± 6.06
		414.11	419.30 ± 10.41	111.25 ± 2.51	2.48	0.22	99.93 ± 3.40	96.87 ± 3.25
	Deacylmetaplexigenin	32.06	32.99 ± 0.82	102.93 ± 2.58	2.51	3.01	99.47 ± 2.82	98.43 ± 4.66
		256.44	250.83 ± 8.59	97.81 ± 3.35	3.42	4.22	99.11 ± 2.74	100.29 ± 3.26
		1923.33	1924.09 ± 66.02	100.04 ± 3.43	3.43	0.46	99.91 ± 4.67	97.20 ± 4.56
Lung	Baishouwu benzophenone	17.57	17.88 ± 1.41	101.78 ± 8.05	7.91	1.58	102.39 ± 5.22	102.64 ± 6.39
		105.43	106.75 ± 4.09	101.25 ± 3.88	3.83	0.17	110.31 ± 8.30	95.70 ± 6.19
		624.26	714.08 ± 15.69	114.39 ± 2.51	2.20	0.46	103.14 ± 0.96	106.73 ± 4.81
	Syringic acid	18.87	20.98 ± 0.81	112.98 ± 4.34	3.84	3.92	110.59 ± 2.05	105.55 ± 11.26
		111.44	99.44 ± 3.12	89.23 ± 2.80	3.14	1.11	89.46 ± 4.21	113.04 ± 6.92
		662.80	694.68 ± 6.54	104.81 ± 0.99	0.94	2.69	103.39 ± 4.31	99.70 ± 3.08
	Qingyangshengenin	10.82	9.31 ± 0.60	86.06 ± 5.56	6.46	2.74	100.97 ± 5.64	100.61 ± 6.46
		64.94	64.83 ± 3.97	99.83 ± 6.11	6.11	1.28	106.71 ± 3.62	95.28 ± 4.30
		384.53	404.73 ± 11.48	105.25 ± 2.99	2.84	2.58	112.33 ± 3.92	92.28 ± 3.92
	Deacylmetaplexigenin	42.31	38.38 ± 2.10	90.72 ± 4.97	5.48	1.60	109.08 ± 5.39	98.24 ± 3.81
		253.84	242.76 ± 11.21	95.64 ± 4.42	4.62	0.23	104.28 ± 3.72	98.27 ± 4.04
		1502.98	1559.69 ± 33.11	103.77 ± 2.20	2.12	1.69	108.85 ± 5.46	94.46 ± 6.23
Kidney	Baishouwu benzophenone	9.84	10.43 ± 0.60	106.05 ± 6.14	5.79	4.71	99.72 ± 9.65	95.86 ± 6.71
		81.96	88.76 ± 6.12	108.29 ± 7.47	6.90	0.66	99.97 ± 6.37	106.47 ± 8.97
		768.40	751.42 ± 2.37	97.80 ± 0.31	0.31	0.70	102.38 ± 1.82	91.67 ± 2.51
	Syringic acid	15.84	17.69 ± 0.69	111.73 ± 4.34	3.89	8.63	88.16 ± 2.64	114.07 ± 4.29
		131.97	129.11 ± 2.81	97.83 ± 2.13	2.18	2.83	99.04 ± 6.71	103.94 ± 4.53
		1242.75	1116.46 ± 5.60	89.84 ± 0.45	0.50	0.84	102.22 ± 6.13	92.51 ± 3.61
	Qingyangshengenin	10.82	11.65 ± 0.74	107.71 ± 6.85	6.36	0.40	97.74 ± 6.19	102.01 ± 10.66
		90.13	96.60 ± 2.48	107.18 ± 2.75	2.57	3.20	94.59 ± 6.15	97.61 ± 6.06
		844.98	878.66 ± 4.64	103.99 ± 0.55	0.53	1.98	103.03 ± 8.87	92.87 ± 1.05
	Deacylmetaplexigenin	28.80	31.53 ± 1.23	109.50 ± 4.26	3.89	1.14	92.94 ± 3.53	90.62 ± 3.28
		239.97	264.80 ± 12.77	110.35 ± 5.32	4.82	0.58	97.37 ± 5.34	107.53 ± 2.81
		2249.71	2276.49 ± 13.14	101.19 ± 0.58	0.58	0.44	101.62 ± 3.50	102.36 ± 5.08
Brain	Baishouwu benzophenone	3.69	4.03 ± 0.04	109.29 ± 1.13	1.03	2.67	100.84 ± 1.48	101.26 ± 0.62
		23.06	23.53 ± 0.49	102.01 ± 2.11	2.06	3.34	101.61 ± 3.22	100.27 ± 2.19
		144.14	137.19 ± 2.44	95.18 ± 1.69	1.78	1.48	98.58 ± 3.75	102.39 ± 4.46
	Syringic acid	8.18	7.63 ± 0.47	93.27 ± 5.72	6.13	4.33	104.48 ± 7.17	102.04 ± 4.88
		51.14	47.56 ± 0.70	92.99 ± 1.37	1.48	4.01	102.55 ± 6.50	102.89 ± 3.95
		321.04	279.18 ± 6.96	86.96 ± 2.17	2.49	1.32	98.13 ± 2.82	100.55 ± 2.44
	Qingyangshengenin	1.51	1.39 ± 0.07	91.78 ± 4.88	5.32	4.70	99.83 ± 7.29	100.00 ± 2.97
		9.47	10.32 ± 0.24	109.02 ± 2.51	2.30	2.28	98.49 ± 2.44	100.34 ± 1.60
		59.16	61.48 ± 0.87	103.92 ± 1.47	1.41	1.90	97.16 ± 2.14	100.57 ± 3.74
	Deacylmetaplexigenin	17.25	16.89 ± 0.38	97.88 ± 2.20	2.25	1.70	99.80 ± 1.14	103.24 ± 1.08
		107.87	110.71 ± 1.90	102.64 ± 1.76	1.72	2.03	102.58 ± 2.61	100.93 ± 1.86
		674.16	648.60 ± 13.75	96.21 ± 2.04	2.12	0.83	99.02 ± 4.12	101.01 ± 1.77
Small intestine	Baishouwu benzophenone	0.46	0.46 ± 0.05	99.09 ± 11.52	11.62	2.54	99.58 ± 8.48	96.59 ± 5.69
		4.97	4.47 ± 0.46	94.60 ± 2.25	10.24	7.25	103.35 ± 4.47	90.73 ± 11.27
		39.22	37.62 ± 3.64	95.91 ± 9.27	9.67	1.20	95.32 ± 7.07	112.25 ± 5.93
	Syringic acid	9.31	9.64 ± 0.99	103.62 ± 10.68	10.31	5.08	96.56 ± 6.17	99.52 ± 8.58
		97.95	103.41 ± 4.57	105.58 ± 4.67	4.42	1.88	96.51 ± 7.72	96.56 ± 10.25
		773.28	818.83 ± 75.08	105.89 ± 10.21	9.65	2.31	89.95 ± 6.78	94.35 ± 10.16
	Qingyangshengenin	20.77	19.50 ± 1.84	93.88 ± 8.88	9.46	3.08	100.93 ± 3.20	98.43 ± 10.45
		218.68	220.60 ± 4.82	100.88 ± 2.21	2.19	5.83	94.35 ± 6.23	95.18 ± 4.67
		1726.39	1873.59 ± 102.17	108.53 ± 5.92	5.45	2.39	90.61 ± 5.32	92.19 ± 11.17
	Deacylmetaplexigenin	14.52	14.03 ± 1.11	96.65 ± 7.64	7.90	3.67	99.24 ± 5.84	98.42 ± 9.24
		152.81	160.30 ± 2.30	104.90 ± 1.51	1.44	3.39	91.30 ± 2.61	93.23 ± 5.55
		1206.41	1272.67 ± 36.89	105.49 ± 3.06	2.90	1.65	91.37 ± 4.67	105.78 ± 6.69
Stomach	Baishouwu benzophenone	0.60	0.59 ± 0.01	98.72 ± 2.01	2.03	6.35	102.37 ± 3.38	101.41 ± 13.01
		6.01	6.06 ± 0.42	100.80 ± 6.91	6.85	1.79	104.40 ± 8.53	93.87 ± 9.89
		45.11	43.35 ± 2.60	96.10 ± 5.76	5.99	2.09	107.00 ± 10.91	95.97 ± 10.79
	Syringic acid	3.44	3.19 ± 0.38	92.78 ± 11.18	12.05	3.44	101.95 ± 11.91	102.90 ± 11.49
		34.37	36.21 ± 1.50	105.37 ± 4.37	4.14	5.62	102.80 ± 2.98	94.74 ± 8.95
		257.76	256.67 ± 15.76	99.58 ± 6.11	6.14	4.52	106.39 ± 7.95	97.95 ± 6.08
	Qingyangshengenin	17.12	17.11 ± 1.56	99.97 ± 9.09	9.10	4.97	96.64 ± 5.92	104.92 ± 11.45
		171.16	196.29 ± 8.27	111.81 ± 3.95	4.21	2.14	98.33 ± 4.86	95.27 ± 2.76
		1283.74	1260.12 ± 70.34	98.16 ± 5.48	5.58	3.07	99.13 ± 2.62	99.66 ± 2.58
	Deacylmetaplexigenin	21.50	21.69 ± 1.83	100.88 ± 8.52	8.44	3.51	97.01 ± 5.95	99.13 ± 4.00
		226.27	250.44 ± 6.48	110.68 ± 2.87	2.59	1.55	99.28 ± 5.10	96.01 ± 1.67
		1786.32	1660.13 ± 76.91	92.94 ± 4.31	4.63	1.88	99.11 ± 3.63	99.62 ± 3.07

**TABLE 3:** Stability of the four compounds in plasma samples. ( $\bar{x}$ ±s, *n* = 5).

Compound	QC (ng·mL^-1^)	Room temperature for 24 h		Three freeze-thaw cycles		4°C for 24 h	
		Measured concentration (ng·mL^-1^)	RSD (%)	Measured concentration (ng·mL^-1^)	RSD (%)	Measured concentration (ng·mL^-1^)	RSD (%)
Baishouwu benzophenone	52.06	55.67 ± 5.93	10.65	56.07 ± 5.75	10.26	55.65 ± 4.01	7.20
	208.25	222.20 ± 17.55	7.90	221.08 ± 17.98	8.13	221.19 ± 11.60	5.24
	1666.00	1664.66 ± 169.47	10.18	1684.19 ± 146.38	8.69	1594.23 ± 107.31	6.73
Deacylmetaplexigenin	49.57	8.67 ± 0.83	6.89	53.08 ± 3.47	6.54	52.01 ± 2.68	5.15
	396.57	60.17 ± 0.96	1.95	352.32 ± 6.40	1.82	406.61 ± 10.59	2.60
	3172.53	489.90 ± 7.17	2.54	3053.63 ± 57.47	1.88	3164.75 ± 84.97	2.68
Qingyangshengenin	8.89	8.67 ± 0.83	9.53	8.91 ± 1.26	14.13	9.19 ± 1.19	12.97
	71.13	60.17 ± 0.96	1.59	64.79 ± 2.45	3.78	63.58 ± 1.58	2.48
	569.17	489.90 ± 7.17	1.46	494.06 ± 14.44	2.92	517.29 ± 16.13	3.12
Syringic acid	1.61	1.55 ± 0.08	5.15	1.42 ± 0.18	12.62	1.47 ± 0.10	6.91
	25.78	23.14 ± 1.80	7.78	23.32 ± 1.20	5.14	22.75 ± 1.62	7.11
	206.21	196.89 ± 6.85	3.48	198.43 ± 6.85	3.45	185.91 ± 1.51	0.81

**TABLE 4:** Stability of the four compounds in tissue homogenate samples ( $\bar{x}$ ±s, *n* = 5).

Matrix	Compound		QC (ng·mL^-1^)	Auto-sampler for 6 h		Three freeze-thaw cycles	
				Measured concentration (ng·mL^-1^)	RSD (%)	Measured concentration (ng·mL^-1^)	RSD (%)
Heart	Baishouwu benzophenone		2.66	2.59 ± 0.05	2.03	2.47 ± 0.03	1.08
			18.96	19.82 ± 0.45	2.27	19.77 ± 0.41	2.05
			111.56	117.44 ± 1.18	1.01	118.10 ± 1.89	1.60
	Syringic acid		8.11	8.45 ± 0.69	8.16	8.60 ± 0.61	7.09
			57.96	48.66 ± 3.17	6.50	51.50 ± 1.66	3.23
			340.93	301.74 ± 5.25	1.74	306.04 ± 11.69	3.82
	Qingyangshengenin		8.35	8.70 ± 0.00	0.00	8.70 ± 0.00	0.00
			59.63	67.73 ± 2.08	3.07	66.54 ± 0.49	0.73
			350.76	394.31 ± 13.51	3.43	400.78 ± 3.50	0.87
	Deacylmetaplexigenin		36.24	29.91 ± 0.63	2.10	30.14 ± 0.33	1.10
			258.89	253.92 ± 7.65	3.01	251.51 ± 4.47	1.87
			1522.88	1545.69 ± 28.28	1.83	1530.72 ± 31.74	2.07
Liver	Baishouwu benzophenone		1.77	1.80 ± 0.19	10.52	1.86 ± 0.05	2.47
			27.19	28.00 ± 1.50	15.67	26.80 ± 1.81	6.75
			313.75	305.22 ± 17.58	5.76	303.56 ± 16.64	5.48
	Syringic acid		22.34	22.04 ± 1.67	7.57	19.70 ± 2.24	11.38
			89.35	85.68 ± 7.15	8.34	83.90 ± 6.79	8.09
			268.07	275.58 ± 10.59	3.84	269.12 ± 16.54	6.14
	Qingyangshengenin		25.26	21.02 ± 1.17	5.58	21.32 ± 1.35	6.33
			386.62	376.28 ± 11.31	3.01	383.88 ± 7.86	2.05
			4484.13	4919.29 ± 148.84	3.02	4962.17 ± 99.47	2.00
	Deacylmetaplexigenin		16.38	15.66 ± 0.68	4.36	15.51 ± 0.68	4.38
			268.09	249.04 ± 7.02	2.83	245.11 ± 4.40	1.79
			3093.35	3350.30 ± 128.16	3.83	3302.83 ± 129.48	4.45
Spleen	Baishouwu benzophenone		10.98	11.24 ± 0.63	5.57	11.40 ± 0.76	6.66
			87.85	80.95 ± 2.54	3.14	79.35 ± 1.81	2.28
			658.88	684.55 ± 31.57	4.61	677.57 ± 32.24	4.76
	Syringic acid		12.54	10.71 ± 0.64	5.95	11.09 ± 0.77	6.97
			100.35	90.24 ± 2.57	2.85	89.84 ± 0.27	0.30
			752.66	709.88 ± 36.02	5.07	691.75 ± 32.30	4.67
	Qingyangshengenin		6.98	6.88 ± 0.56	8.13	7.15 ± 0.84	11.72
			55.80	53.51 ± 1.93	3.61	52.30 ± 1.27	2.42
			418.52	424.65 ± 113.39	3.15	420.89 ± 15.01	3.57
	Deacylmetaplexigenin		33.00	31.95 ± 1.48	4.63	32.15 ± 1.58	4.92
			263.97	232.19 ± 3.53	1.52	247.56 ± 6.23	2.52
			1979.74	1990.73 ± 48.18	2.49	1950.06 ± 62.22	3.19
Lung	Baishouwu benzophenone		17.66	17.71 ± 1.84	10.40	17.55 ± 2.16	12.30
			105.98	107.37 ± 5.82	5.42	105.63 ± 6.43	6.09
			630.29	717.67 ± 14.53	2.03	716.31 ± 12.93	1.81
	Syringic acid		18.57	20.69 ± 0.65	3.13	19.69 ± 0.70	3.58
			111.44	99.02 ± 4.40	4.45	99.65 ± 4.46	4.47
			662.80	699.44 ± 16.38	2.34	690.17 ± 22.43	3.25
	Qingyangshengenin		10.81	9.77 ± 0.72	7.33	9.71 ± 0.57	5.90
			64.88	66.10 ± 3.48	5.27	65.51 ± 2.87	4.39
			385.86	402.91 ± 10.73	2.66	398.02 ± 4.47	1.12
	Deacylmetaplexigenin		42.42	38.33 ± 2.37	6.19	37.62 ± 2.38	6.33
			254.52	243.55 ± 12.09	4.96	240.11 ± 10.98	4.57
			1513.71	1544.16 ± 36.02	2.32	1549.77 ± 41.79	2.70
Kidney	Baishouwu benzophenone		9.84	9.74 ± 0.44	4.52	9.58 ± 0.63	6.54
			81.96	88.88 ± 5.31	5.98	87.75 ± 5.24	5.97
			768.40	750.99 ± 14.44	1.92	748.36 ± 12.9	1.72
	Syringic acid		15.75	15.16 ± 0.43	2.81	14.45 ± 0.74	5.15
			131.25	120.71 ± 4.79	3.97	116.78 ± 6.43	5.51
			1230.44	1055.65 ± 22.26	2.11	1075.85 ± 6.57	0.61
	Qingyangshengenin		10.82	11.57 ± 0.47	4.05	11.81 ± 0.59	5.01
			90.13	101.23 ± 2.63	2.60	101.81 ± 0.94	0.92
			844.98	912.57 ± 17.67	1.94	899.11 ± 32.81	3.65
	Deacylmetaplexigenin		28.80	30.96 ± 0.89	2.86	30.51 ± 1.21	3.97
			239.97	267.87 ± 13.29	4.96	266.54 ± 9.78	3.67
			2249.71	2268.44 ± 53.12	2.34	2270.72 ± 48.43	2.13
Brain	Baishouwu benzophenone		3.34	3.08 ± 0.01	0.24	3.02 ± 0.04	1.33
			20.88	19.76 ± 0.44	2.25	20.10 ± 0.34	1.69
			131.10	120.63 ± 2.55	2.12	123.76 ± 3.01	2.43
	Syringic acid		8.18	7.01 ± 0.09	1.23	7.03 ± 0.24	3.38
			51.14	44.40 ± 1.20	2.71	45.14 ± 1.38	3.06
			320.04	283.47 ± 6.56	2.32	287.12 ± 6.21	2.16
	Qingyangshengenin		1.58	1.57 ± 0.05	3.49	1.52 ± 0.05	3.47
			9.90	11.09 ± 0.23	2.07	11.11 ± 0.06	0.52
			62.12	65.57 ± 1.12	1.71	67.29 ± 1.69	2.51
	Deacylmetaplexigenin		17.26	16.37 ± 0.20	1.21	16.11 ± 0.43	2.67
			107.87	106.81 ± 0.35	0.33	107.57 ± 1.64	1.52
			677.19	639.39 ± 12.53	1.96	657.30 ± 18.60	2.83
Small intestine	Baishouwu benzophenone		0.46	0.48 ± 0.05	9.78	0.46 ± 0.04	7.90
			4.97	4.71 ± 0.14	3.07	4.66 ± 0.10	2.24
			39.22	38.84 ± 3.48	8.97	38.30 ± 3.03	7.90
	Syringic acid		9.31	9.13 ± 1.24	13.60	9.19 ± 1.54	16.72
			97.95	100.06 ± 2.52	2.52	103.28 ± 6.88	6.67
			773.28	851.12 ± 6.95	0.82	846.13 ± 38.39	4.54
	Qingyangshengenin		20.77	18.85 ± 1.77	9.39	19.05 ± 1.38	7.27
			218.68	197.14 ± 6.20	3.15	207.46 ± 2.44	1.17
			1726.39	1879.63 ± 80.93	4.31	1835.86 ± 71.32	3.88
	Deacylmetaplexigenin		14.52	12.77 ± 1.27	9.97	12.39 ± 1.33	10.73
			152.81	150.09 ± 2.73	1.82	154.01 ± 1.23	0.80
			1206.41	1293.63 ± 65.40	5.06	1296.24 ± 110.99	8.56
Stomach	Baishouwu benzophenone		0.63	0.57 ± 0.05	9.09	0.58 ± 0.07	11.47
			6.28	6.13 ± 0.11	1.77	6.22 ± 0.15	2.41
			47.06	43.94 ± 1.72	3.91	44.41 ± 1.38	3.10
	Syringic acid		3.44	3.19 ± 0.42	13.31	3.28 ± 0.44	13.37
			34.37	32.61 ± 0.71	2.17	34.03 ± 0.70	2.06
			257.76	237.17 ± 11.53	4.86	240.90 ± 12.79	5.31
	Qingyangshengenin		17.14	15.93 ± 1.17	7.32	17.23 ± 1.91	11.08
			171.39	193.21 ± 6.26	3.24	188.55 ± 4.07	2.16
			1285.45	1185.09 ± 69.62	5.88	1245.72 ± 61.42	4.93
	Deacylmetaplexigenin		22.21	21.14 ± 1.50	7.10	21.58 ± 1.95	9.04
			222.09	244.68 ± 7.59	3.10	245.34 ± 9.19	3.75
			1665.65	1622.87 ± 76.11	4.69	1662.82 ± 50.13	3.01

### 3.2 PK study

**FIGURE 2:**
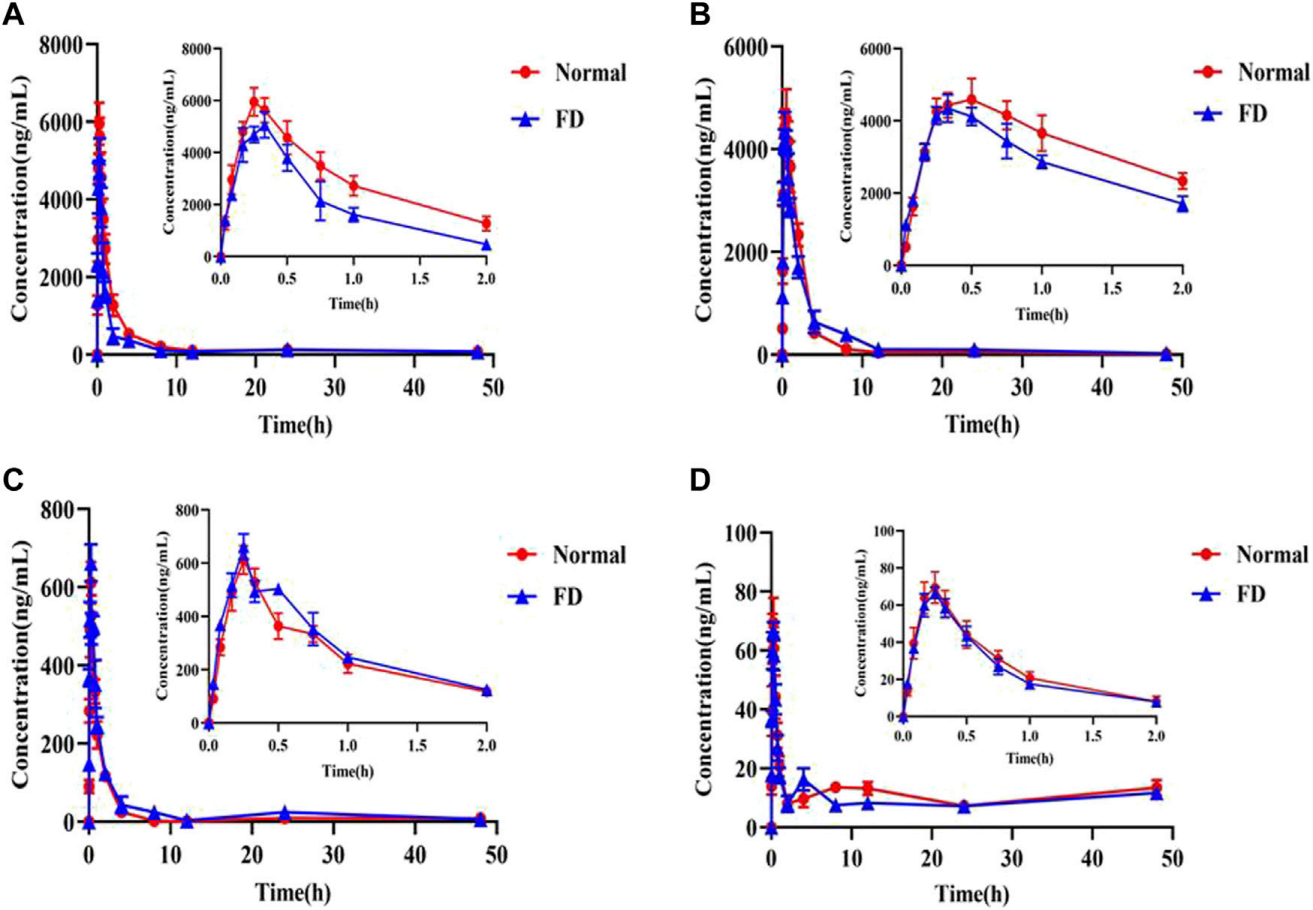
Concentration-time profiles of the four index compositions after oral administration of the CA extract in plasma samples ( $\bar{x}$ ±SD, *n* = 6). **(A)** Baishouwu benzophenone **(B)** Deacylmetaplexigenin **(C)** Qingyangshengenin **(D)** Syringic acid.

The validated method was successfully applied to determine the plasma concentration of orally administered CA extract in the normal and FD groups at each time point. The mean plasma concentration–time curves are presented in Figure 2, and PK parameters are listed in Table 5. The results demonstrated that the time of peak concentration (T_max_) was approximately 15 min for all components, except deacylmetaplexigenin (T_max_: 20–30 min), and half–life (T_1/2_) of the four components was more than 9 h, indicating that four components had rapid absorption and slow elimination. Compared with the normal group, the FD group had higher T_max_, half–life (T_1/2_), and plasma clearance (CL/F) of baishouwu benzophenone but significantly lower peak concentration (C_max_) and the area under the plasma concentration–time curve from time zero to time t (AUC_0-t_). This indicated that FD is associated with lower absorption and increased clearance of baishouwu benzophenone. In contrast, the T_1/2_ and AUC_0-t_ (*p* < 0.05) of deacylmetaplexigenin and qingyangshengenin were higher in the FD group than in the normal group. Both better absorption (higher AUC and C_max_) and slower elimination rate (higher T_1/2_) contribute to higher bioavailability; as a result, the bioavailability of deacylmetaplexigenin and qingyangshengenin in CA was improved in the pathological state. Meanwhile, the lower CL/F (*p* < 0.05) of qingyangshengenin was observed in FD group, illustrating qingyangshengenin have the potential to accumulate in tissues. The FD group had higher T_max_ and T_1/2_ of syringic acid but lower C_max_ and AUC_0-t_ than the normal group, albeit without significant differences. The above results showed that the exposure of baishouwu benzophenone and deacylmetaplexigenin in normal and FD rats was much higher than that of qingyangshengenin and syringic acid, and the pathological state could increase the exposure of deacylmetaplexigenin and qingyangshengenin and decrease the exposure of baishouwu benzophenone in rats.

**TABLE 5:** The pharmacokinetic parameters of the four compositions after oral administration of CA extract in rats plasma samples ( $\bar{x}$ ±SD, *n* = 6).

Pharmacokinetic parameters	Baishouwu benzophenone		Deacylmetaplexigenin		Qingyangshengenin		Syringic acid	
	Normal group	FD group	Normal group	FD group	Normal group	FD group	Normal group	FD group
T_max_ (h)	0.26 ± 0.03	0.31 ± 0.06	0.42 ± 0.11	0.33 ± 0.08	0.25 ± 0.08	0.25 ± 0.05	0.24 ± 0.03	0.25 ± 0.04
C_max_ (µg/L)	6004.45 ± 592.01	5218.08 ± 440.24^*^	4679.95 ± 499.39	4509.98 ± 241.37	611.37 ± 53.33	662.44 ± 47.65	72.20 ± 6.74	67.38 ± 3.00
T_1/2_ (h)	9.14 ± 2.41	10.97 ± 1.85	10.03 ± 3.16	12.86 ± 7.56	9.23 ± 2.25	11.73 ± 7.60	34.52 ± 6.73	37.68 ± 12.61
AUC_0-t_ (h·µg/L)	12,797.64 ± 911.28	9822.96 ± 1295.73^***^	11,599.44 ± 1305.50	13,262.37 ± 658.39^*^	986.02 ± 149.76	1609.29 ± 525.84^*^	523.26 ± 54.26	497.89 ± 109.02
AUC_0-∞_ (h·µg/L)	13,798.83 ± 1267.10	11,104.95 ± 1664.93^**^	12,255.16 ± 2468.74	13,908.39 ± 1073.84	1205.20 ± 453.77	2677.51 ± 2513.92	1152.53 ± 152.98	1166.16 ± 357.26
CL/F (L/h/kg)	0.36 ± 0.03	0.45 ± 0.07^**^	1.15 ± 0.20	0.99 ± 0.07	8.73 ± 2.11	5.66 ± 2.56^*^	0.10 ± 0.01	0.11 ± 0.04
Vz/F (L/kg)	4.67 ± 0.88	7.08 ± 1.07^*^	16.02 ± 3.79	18.07 ± 10.56	110.81 ± 13.91	70.19 ± 14.07^***^	5.07 ± 0.74	5.49 ± 0.69

*P < 0.05. **p < 0.01, and ***p < 0.001 *versus* normal group.

### 3.3 Tissue distribution study

**FIGURE 3:**
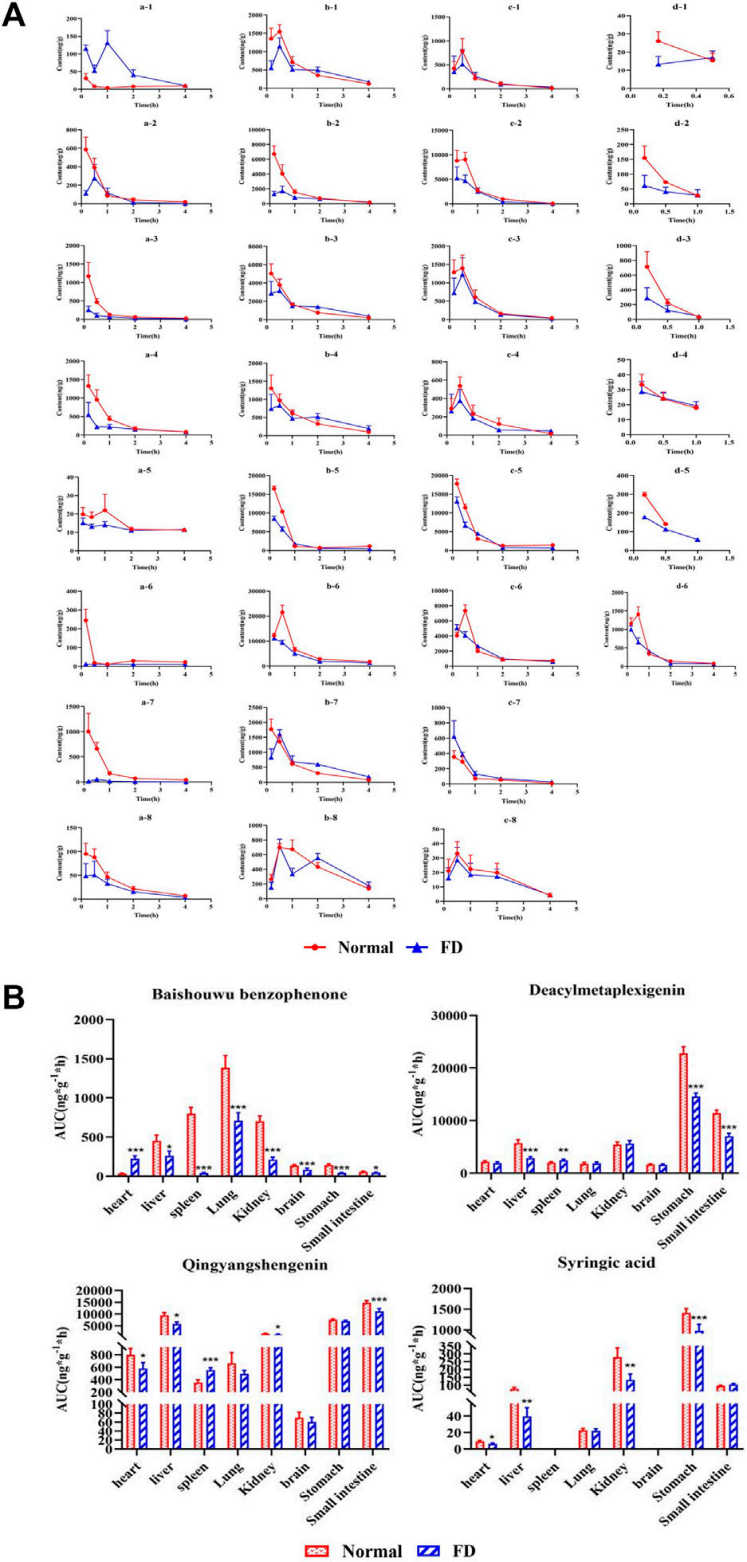
The tissue distribution profiles **(A)** and AUC_0-t_ **(B)** of the four compositions of CA extract in normal and FD rats ( $\bar{x}$ ±s, *n* = 6): (a) Baishouwu benzophenone, (b) Deacylmetaplexigenin, (c) Qingyangshengenin, (d) Syringic acid; heart (1), liver (2), kidney (3), lung (4), small intestine (5), stomach (6), spleen (7), brain (8). ^*^ *p* < 0.05, ^**^ *p* < 0.01, and ^***^ *p* < 0.001 *versus* the normal group.

After oral administration, drugs are absorbed into the blood and delivered to organs via blood circulation. To further explore the distribution of baishouwu benzophenone, deacylmetaplexigenin, qingyangshengenin, and syringic acid *in vivo*, their tissue distributions were assessed. As shown in Figures 3A, four components were widely distributed in various tissues, including the heart, liver, spleen, lung, kidney, stomach, small intestine, and brain. The AUC_0-t_ of four components is exhibited in Figures 3B. Baishouwu benzophenone, deacylmetaplexigenin, and qingyangshengenin peaked at 0.17–0.5 h in most of organ tissues and were almost completely metabolized at 4 h after oral administration, whereas syringic acid in all tissues was eliminated 1 h after dosing, showing no long–term accumulation of these components as exhibited in Figures 3A. Compared with that in the normal group, the concentration of baishouwu benzophenone in the FD group was the highest in the lungs, followed by that in the liver, heart, kidney, brain, small intestine, spleen, and stomach, suggesting that the lungs might be the main organs for the accumulation of baishouwu benzophenone as shown in Figures 3B. In the FD group, the AUC_0-t_ of baishouwu benzophenone was significantly decreased in all tissues, except in the heart (*p* < 0.05). The concentration of deacylmetaplexigenin and qingyangshengenin in the FD group was the highest in the stomach and small intestine, followed by that in the kidney and liver. The AUC_0-t_ of deacylmetaplexigenin and qingyangshengenin was significantly decreased in the liver, stomach and small intestine of the FD group than the normal group (*p* < 0.001), indicating that deacylmetaplexigenin and qingyangshengenin has a good affinity in the gastrointestinal region. The results were consistent with those of the previous studies, which showed that these four components were absorbed to a certain extent in the whole intestinal segment and had good intestinal affinity (Liu et al., 2022; Sun et al., 2022). In addition, the concentrations of deacylmetaplexigenin and qingyangshengenin were lower in some tissues with abundant blood flow, such as brain, heart, liver, and kidney, indicating the blood flow of tissue had little effect on distribution of deacylmetaplexigenin and qingyangshengenin. However, the distribution of deacylmetaplexigenin and qingyangshengenin in the brain indicates that the drug may pass through the blood–brain–barrier. Syringic acid had higher distribution in the stomach. Of note, syringic acid was not detected in the brain, which appeared to be due to its low concentration or its poor ability to cross the blood–brain–barrier. The AUC_0-t_ of syringic acid was significantly lower in all tissues, except the lung and small intestine, in the FD group vs the normal group (*p* < 0.05). The above results suggesting that the four components of CA extract were effectively distributed to the gastrointestinal tract.

## 4 Discussion

UPLC–MS/MS allows the separation, qualitative analysis, and quantitative analysis of complex compounds, owing to which it is an important method for determining the active components in TCM molecules and compound formulations. In addition, UPLC–MS/MS is characterized by accurate quantification, quick analysis, and high sensitivity. It can avoid matrix interference during the analysis and simultaneously detect multiple components in the sample (Qin et al., 2021b; Jing, 2023). In terms of sample preparation, we investigated the effects of liquid–liquid extraction (using ethyl acetate and n–butanol) and protein precipitation using organic solvents (methanol and acetonitrile) on the pretreatment and detection of drug–containing plasma. Additionally, the analytes to be quantified (acetophenone, C21 steroids, and phenolic acid compounds) are weakly acidic compounds, and these exist in a non–dissociated state under acidic conditions. The finally determined plasma processing method involved acidification with 1% formic acid, followed by protein precipitation with acetonitrile and immediate vortexing to prevent protein encapsulation of the analytes. With this method, the extraction recovery of the four CA components from plasma samples was within an acceptable range, and no matrix interference was observed. Moreover, the accuracy and intra–day and inter–day precision indicated good repeatability and high reliability of the method, meeting the requirements for the analysis of biological samples.

Recently, several studies have indicated that the PK characteristics of TCM compounds are different under physiological and pathological conditions. This is primarily attributed to the changes in drug metabolism enzymes, drug transporters, membrane permeability, and gut microbiota. All of these factors can affect the absorption, distribution, metabolism, and excretion of drugs *in vivo* (Gong et al., 2015). Specifically, in patients with FD, inflammation can alter cell permeability and expression of transporters on cell membranes, leading to changes in the PK parameters of drugs, which ultimately affects their therapeutic efficacy (Gong et al., 2009; Alexander et al., 2012). The present study found that the total absorption of baishouwu benzophenone and syringic acid was lower in FD rats than in normal rats, with a shorter duration (higher CL/F). In contrast, deacylmetaplexigenin and qingyangshengenin had higher absorption in FD rats than in normal rats, albeit with a longer duration (lower CL/F). Therefore, it is speculated that in patients with FD, differences in the PK parameters of CA components may be related to alterations, such as gastrointestinal dysfunction, imbalanced intestinal flora, and altered hepatic enzymes, which can alter drug absorption and metabolism.

In this animal study, significant differences were observed in the distribution of qingyangshengenin in various tissues between normal and FD rats. Compared with the normal group, the FD group had lower AUC_0-t_ of the four components in tissues, except for baishouwu benzophenone in the heart, qingyangshengenin and deacylmetaplexigenin in the spleen, and deacylmetaplexigenin in the lungs and kidneys. This suggests that the pathological state of FD can affect the distribution of drugs in tissues. Reportedly, the differential distribution of drugs mainly depends on blood flow, organ perfusion rate, and affinity of drugs (Amandla et al., 2016). On the contrary, the absorption of orally administered drugs is influenced by factors such as the pH of gastrointestinal fluids, gastric emptying, gastric transit rate, intestinal motility, gastrointestinal metabolism, and gastrointestinal diseases. Studies have shown that delayed or accelerated intestinal motility and hypersensitivity reactions are the major mechanisms underlying the pathophysiology of FD (Hidekazu, 2020). In addition, impaired gastric emptying and reduced gastric motility may decrease drug absorption, thereby reducing the concentration of drugs entering tissues in pathological states.

Existing studies have shown that one of the important factors causing FD is impaired gastrointestinal motility, which results from malfunctioning of the enteroendocrine cells and enteric nervous system. Gastric acid secretion (GAS) and motilin (MTL) are both gastrointestinal hormones that regulate gastrointestinal motility. GAS is mainly secreted by G cells and is mostly present in the gastric antrum, gastric fundus, and proximal duodenal mucosa. It is regulated by vagal nerve excitation and promotes the secretion of hydrochloric acid and pepsinogen, protects the gastric and intestinal mucosa, and enhances gastrointestinal motility. MTL is secreted by Mo cells and acts directly on specific receptors in the gastric smooth muscle, releasing acetylcholine, promoting strong gastric contractions and small intestinal segmentation movements, and facilitating gastric emptying (Du et al., 2020; Li et al., 2021; Zhao and Zhang, 2021). Studies have demonstrated decreased levels of the gastrointestinal motility hormones GAS and MTL in patients with FD (Li et al., 2001; Liang et al., 2021). The present study found that CA components, such as qingyangshengenin and deacylmetaplexigenin are present at high concentrations in the stomach and small intestine of both normal and FD rats. Based on the theory that the target organs may be the primary sites for pharmacological effects, we speculate that these components may act on the stomach and small intestine to regulate GAS and MTL levels, further enhancing their anti-FD effects.

Further advancements in the pathogenesis of FD have revealed that psychosocial factors additionally play an important role in disease development. According to the Rome IV criteria, FD is a disorder of the gut–brain axis. Reportedly, the gut–brain interaction is mediated via a bidirectional neuroendocrine network between the brain and the gut. Patients with FD often experience varying degrees of psychological disorders such as depression, anxiety, and somatization. In addition, psychological factors such as anxiety and depression also act on the gut–brain axis. Dysfunction of this axis can lead to abnormal expression of gut–brain peptides, including gastrointestinal hormones, gastrointestinal neuropeptides, and neurotransmitters. In particular, changes in gastrointestinal hormone levels are the most significant in the pathological states related to gut–brain peptides. Moreover, abnormal expression of gut–brain peptides is closely related to gastrointestinal motility disorders. The gut–brain axis participates in the pathogenesis of FD by regulating the levels of the gut–brain peptides (Douglas and William, 2016; Wang et al., 2017; Mao et al., 2021). Therefore, brain tissue may be another target organ of CA for the treatment of FD. It is speculated that the CA components qingyangshengenin, deacylmetaplexigenin, and baishouwu benzophenone can cross the blood–brain barrier, which indicates their potential to alter the levels of gastrointestinal hormones (MTL and GAS) and thus regulate the gut–brain axis and exert anti–FD effects.

## 5 Conclusion

In this study, a sensitive, rapid, and reliable UPLC–ESI–MS/MS method was established, validated, and applied for the quantification of four active components in CA extract in plasma and tissue samples of normal and FD rats. After oral administration, the active components of CA (qingyangshengenin, baishouwu benzophenone, deacylmetaplexigenin, and syringic acid) were quickly absorbed and widely distributed in various tissues. The deacylmetaplexigenin and qingyangshengenin had higher absorption and lower elimination in the FD group, which indicated higher accumulation of the components in target organs for anti–FD effects. However, the baishouwu benzophenone had lower absorption and higher elimination in the FD group. Furthermore, the volume of distribution of qingyangshengenin, deacylmetaplexigenin, and syringic acid was high in the stomach and small intestine, indicating that these could be the main components mediating the pharmacodynamics of FD. In addition, the brain may be another target organ of CA for the treatment of FD, as the gut–brain axis can influence the pathogenesis of FD. The four components may alter gastrointestinal hormone levels by regulating the gut-brain axis to mediate an anti–FD effect. Collectively, the findings from this study provide valuable insight into the PK and distribution parameters of CA. Further studies are required to explore the differences in the PK and tissue distribution parameters of the active components of CA based on transport route and metabolism under physiological and pathological states.

## Data Availability

The original contributions presented in the study are included in the article/Supplementary Material, further inquiries can be directed to the corresponding authors.

## Abbreviations

AUC_0-t_, the area under the plasma concentration–time curve from time zero to time t; AUC_0-m_, the area under the plasma concentration-time curve from time zero to time infinity; CA, *Cynanchum auriculatum*; CL/F, plasma clearance; C_max_, maximum concentration; ESI, electrospray ionization; FD, functional dyspepsia; FDA, the Food and Drug Administration; GAS, Gastric acid secretion; IS, internal standard; MRM, multiple reaction monitoring; MTL, motilin; NMPA, National Medical Products Administration; PK, pharmacokinetic; QC, quality control; *R* ^*2*^, correlation coefficient; RSD, relative standard deviation; SD, standard deviation; T_1/2_, elimination half–life; TCM, traditional Chinese medicine; T_max_, the time of peak concentration; UPLC–MS/MS, Ultra–performance liquid chromatography coupled with triple quadrupole mass spectrometry; Vz/F, apparent volume of distribution; *1/X* ^*2*^, linear weighted.
