# Supplementary material for: Pharmacokinetics and tissue distribution of four major bioactive components of Cynanchum auriculatum extract: a UPLC–MS/MS study in normal and functional dyspepsia rats
Source: Front Pharmacol. 2023 Oct 17;14:1279971. doi: 10.3389/fphar.2023.1279971 (PMC10616469; doi:10.3389/fphar.2023.1279971)
Supplement: Supplementary file 1 [file DataSheet1.docx]

Supplementary Material

Pharmacokinetics and tissue distribution of four major bioactive components of *Cynanchum auriculatum* extract: A UPLC–MS/MS study in normal and functional dyspepsia rats

**List of Supplementary Material Captions**

**Supplementary Figure S1.** Representative chromatograms. (A) blank plasma or tissue homogenate samples; (B) blank plasma or tissue homogenate samples spiked with the four components and IS; (C) plasma and tissue homogenate samples after oral administration of CA extract. (a) Plasma, (b) Heart, (c) Liver, (d) Kidney, (e) Lung, (f) Stomach, (g) Small intestine, (h) Spleen, (i) Brain. (1. qingyangshengenin; 2. puerarin; 3. deacylmetaplexigenin; 4. baishouwu benzophenone; 5.syringic acid)


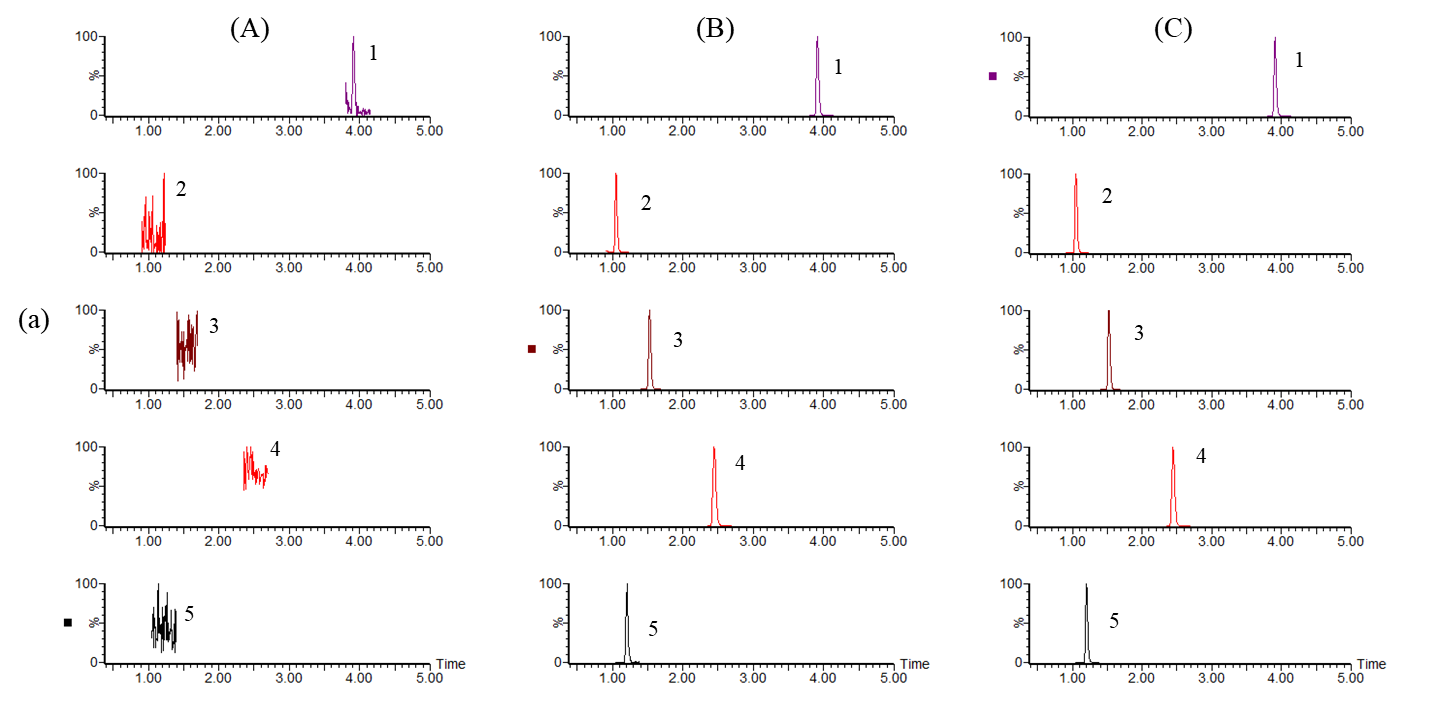

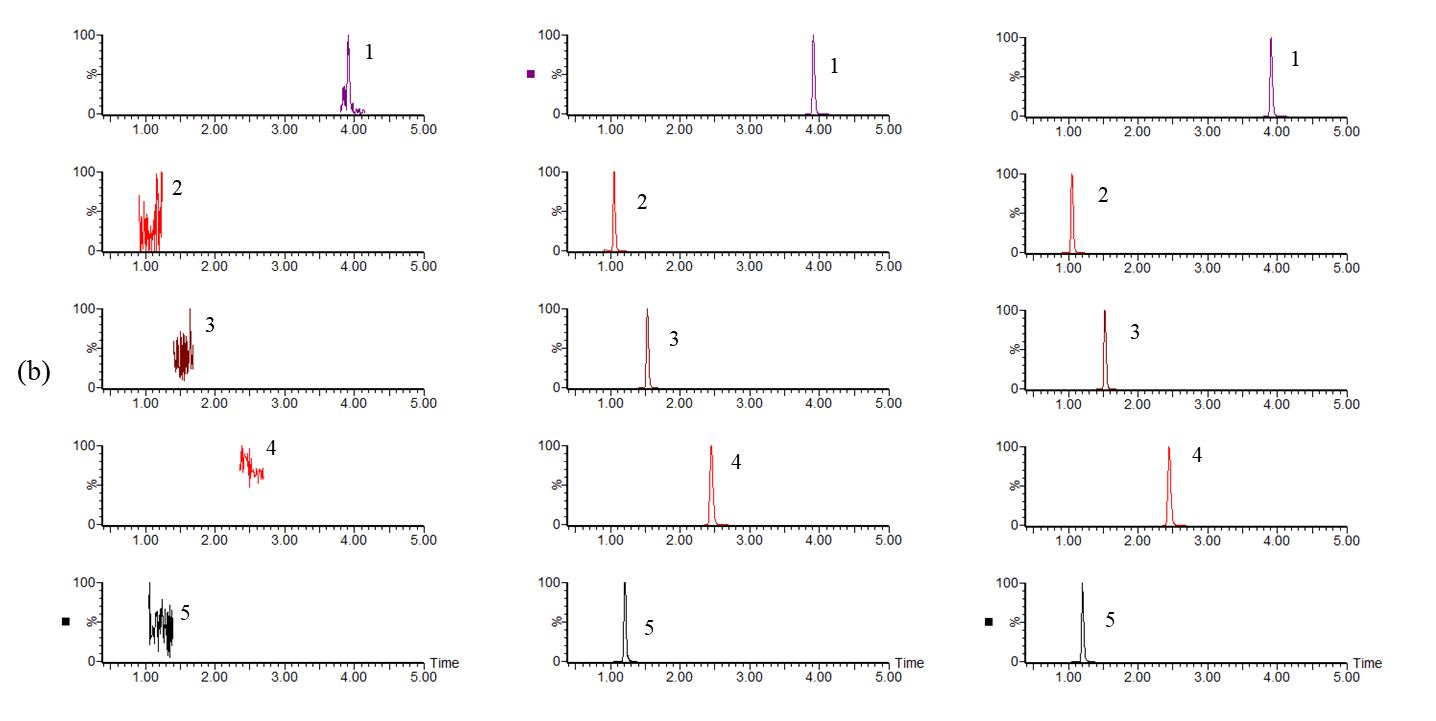

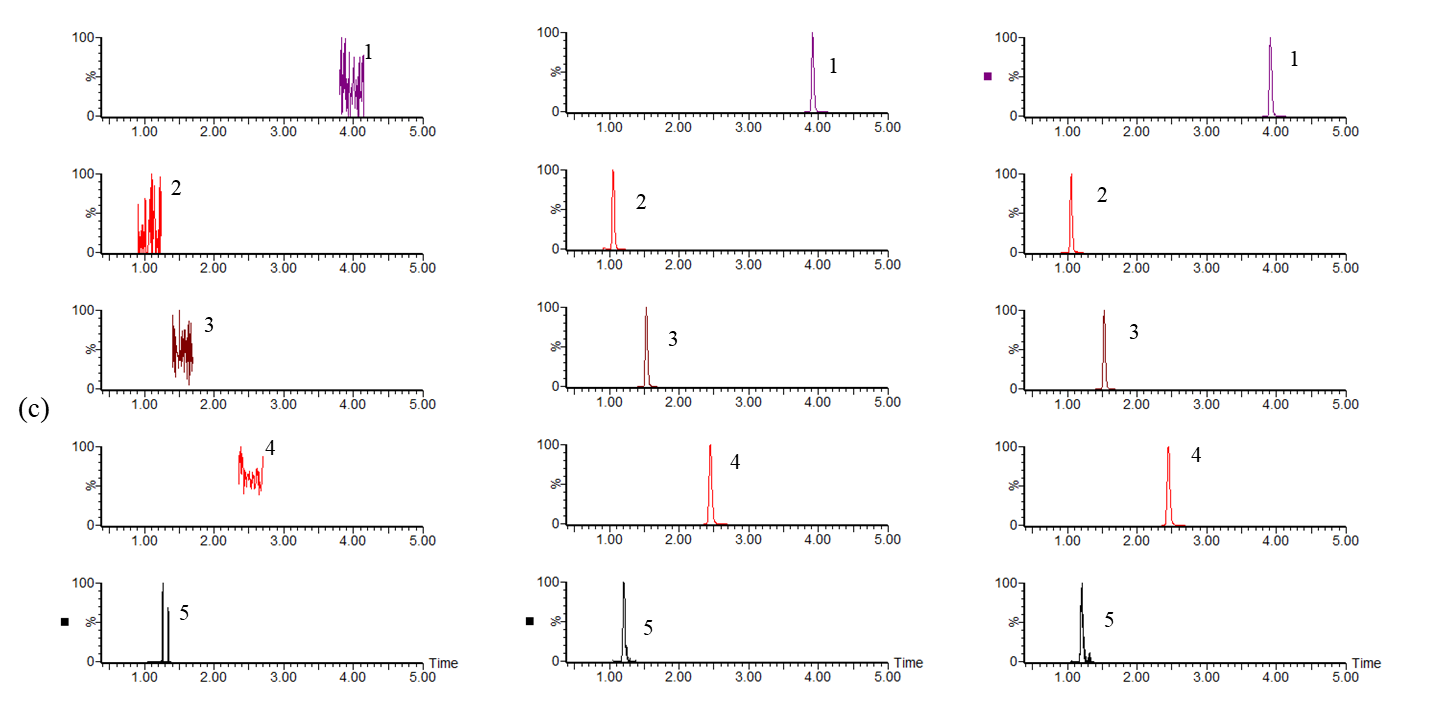

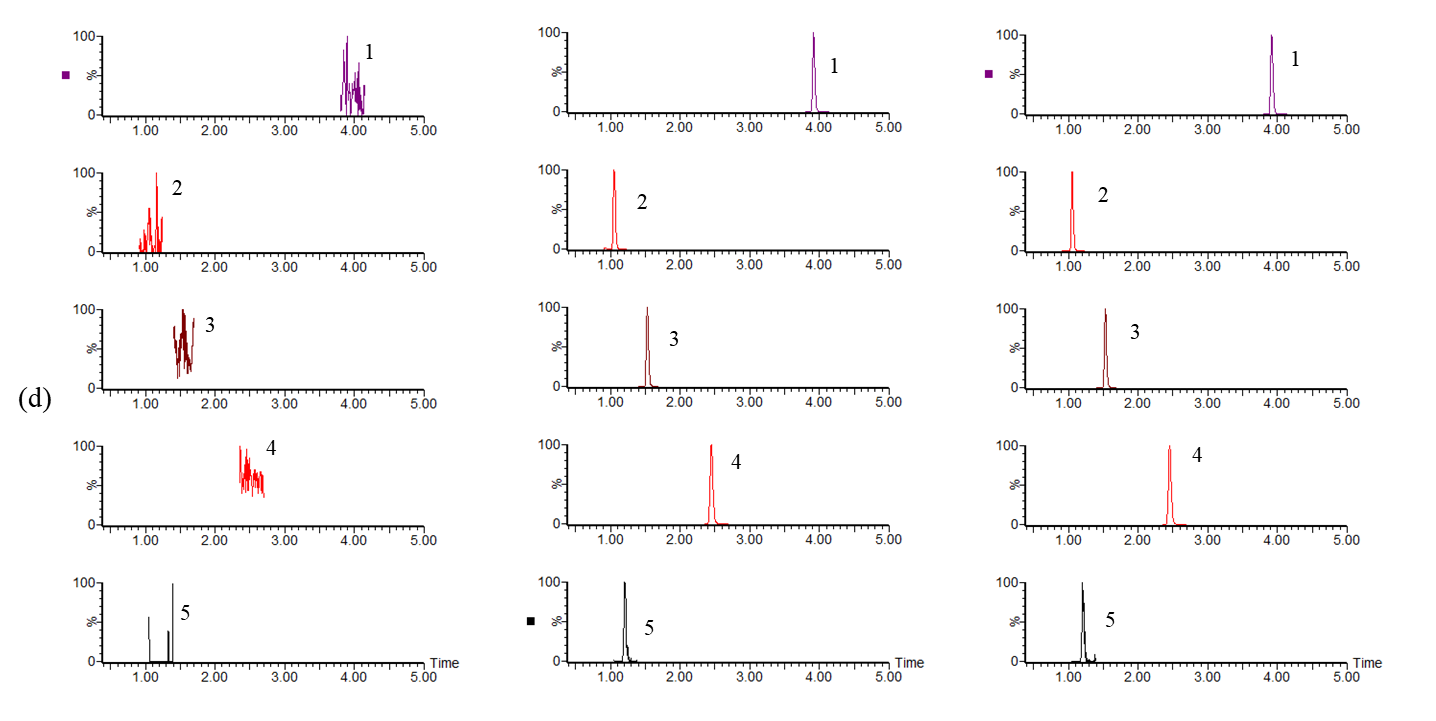

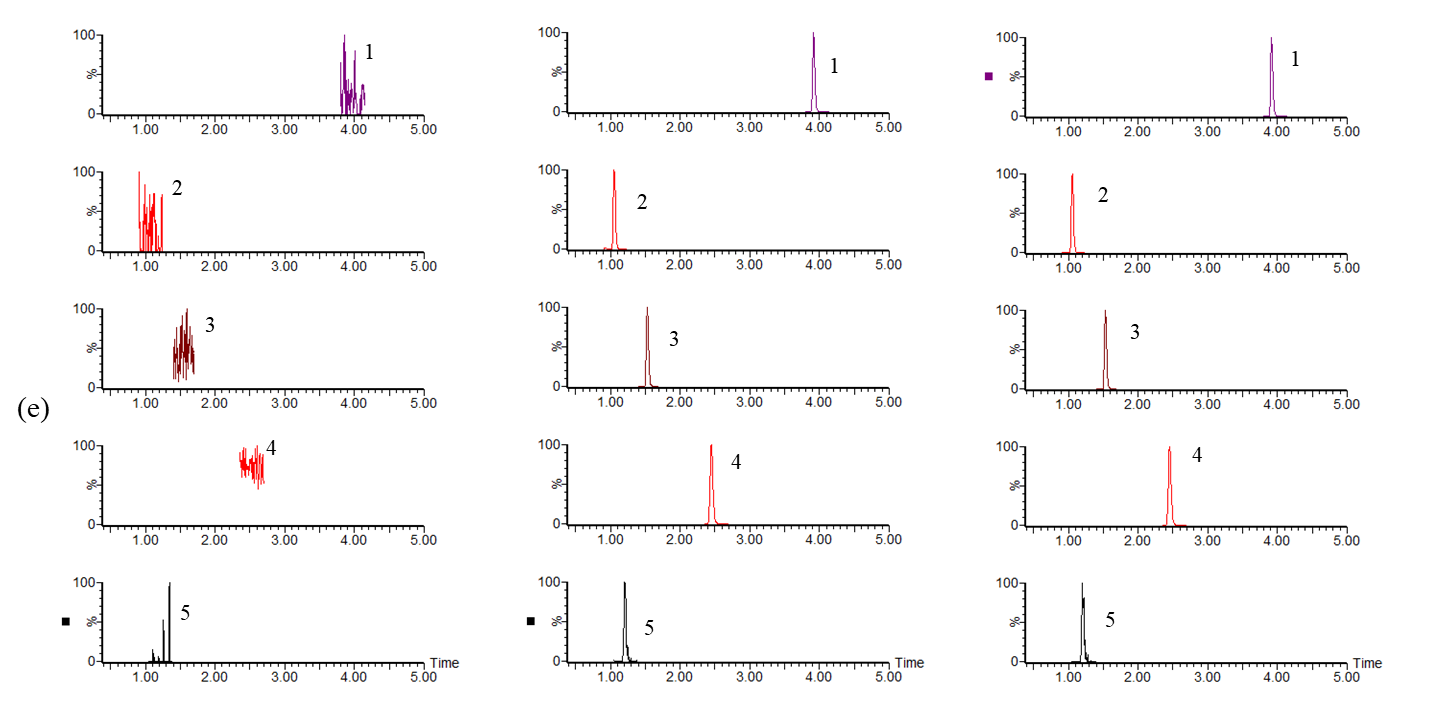

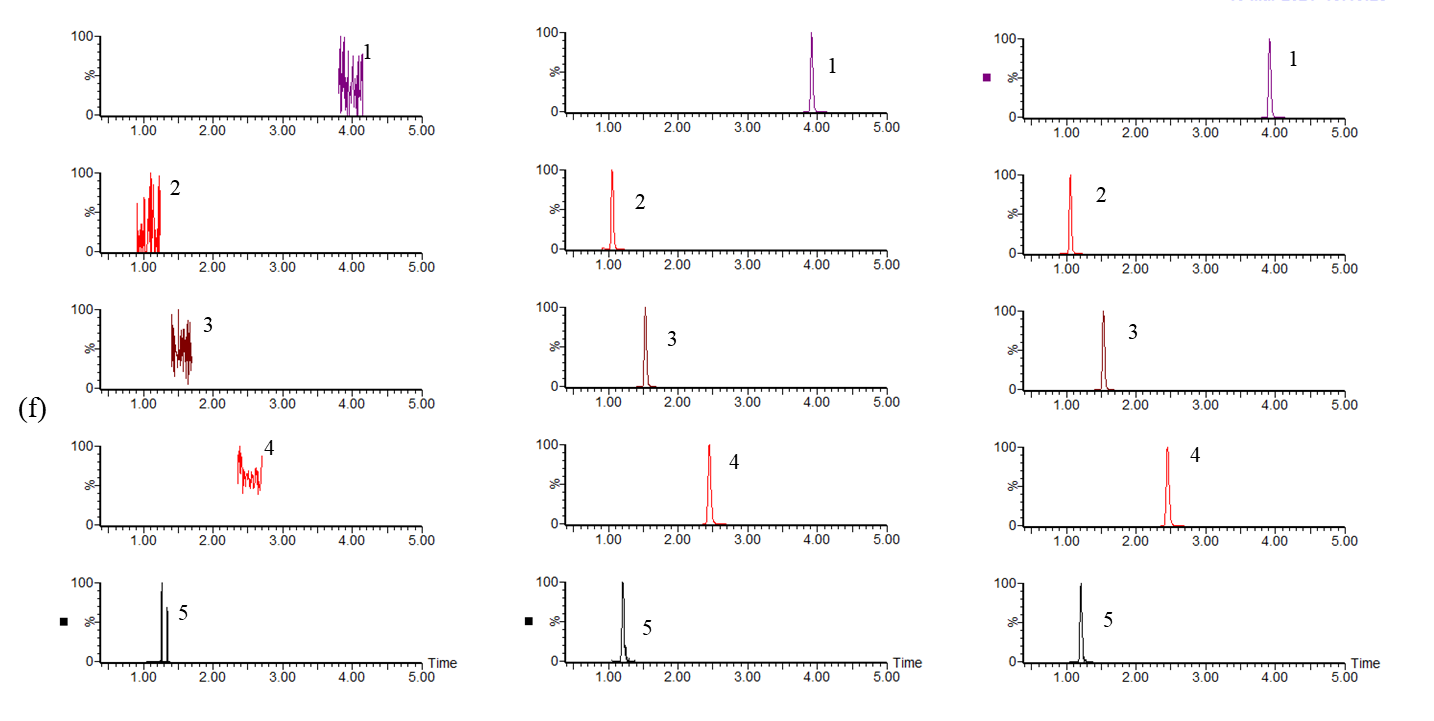

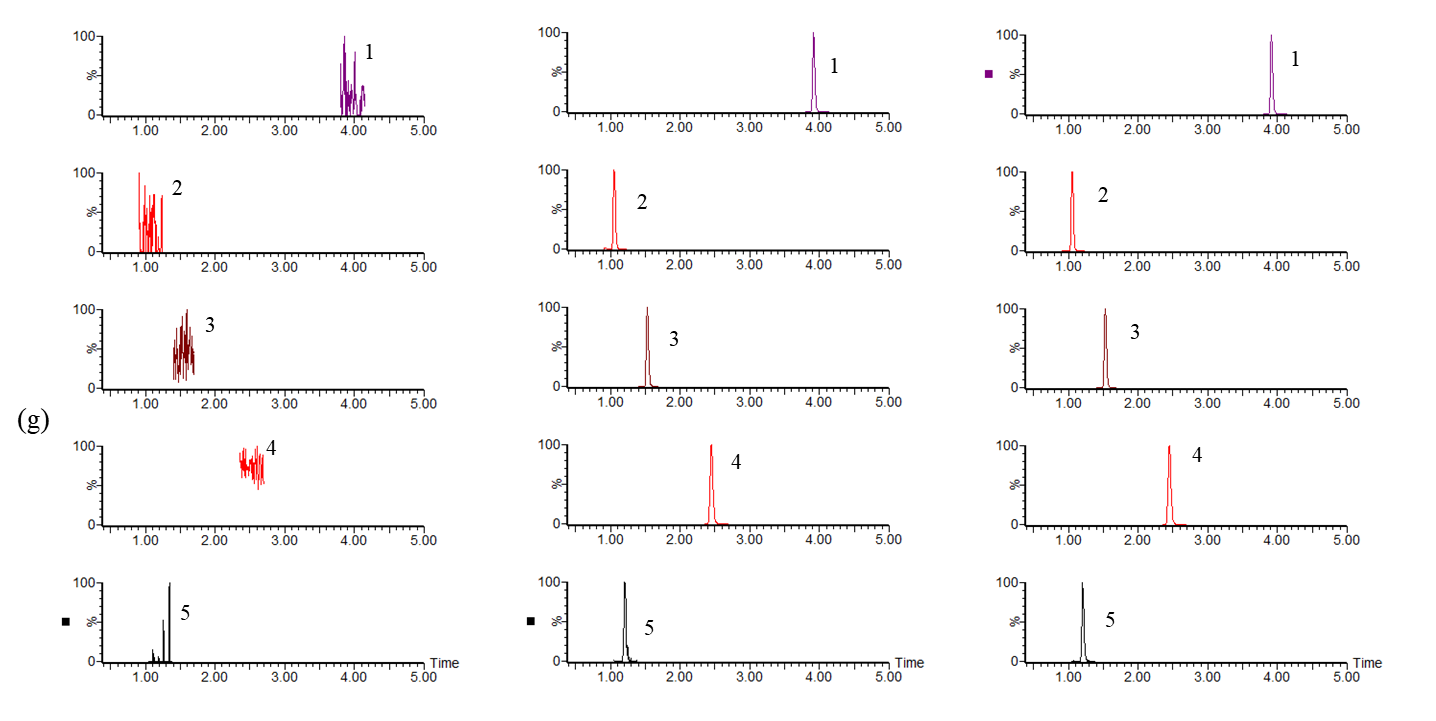

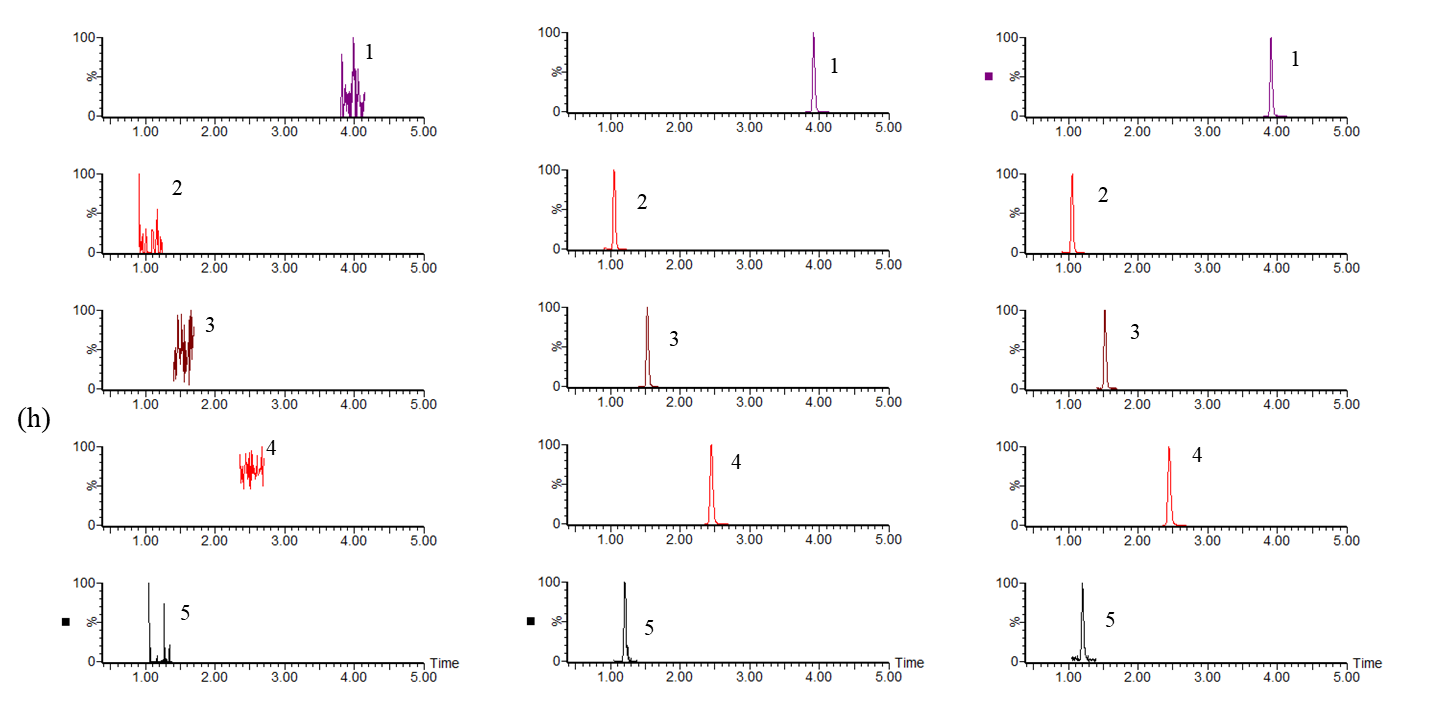

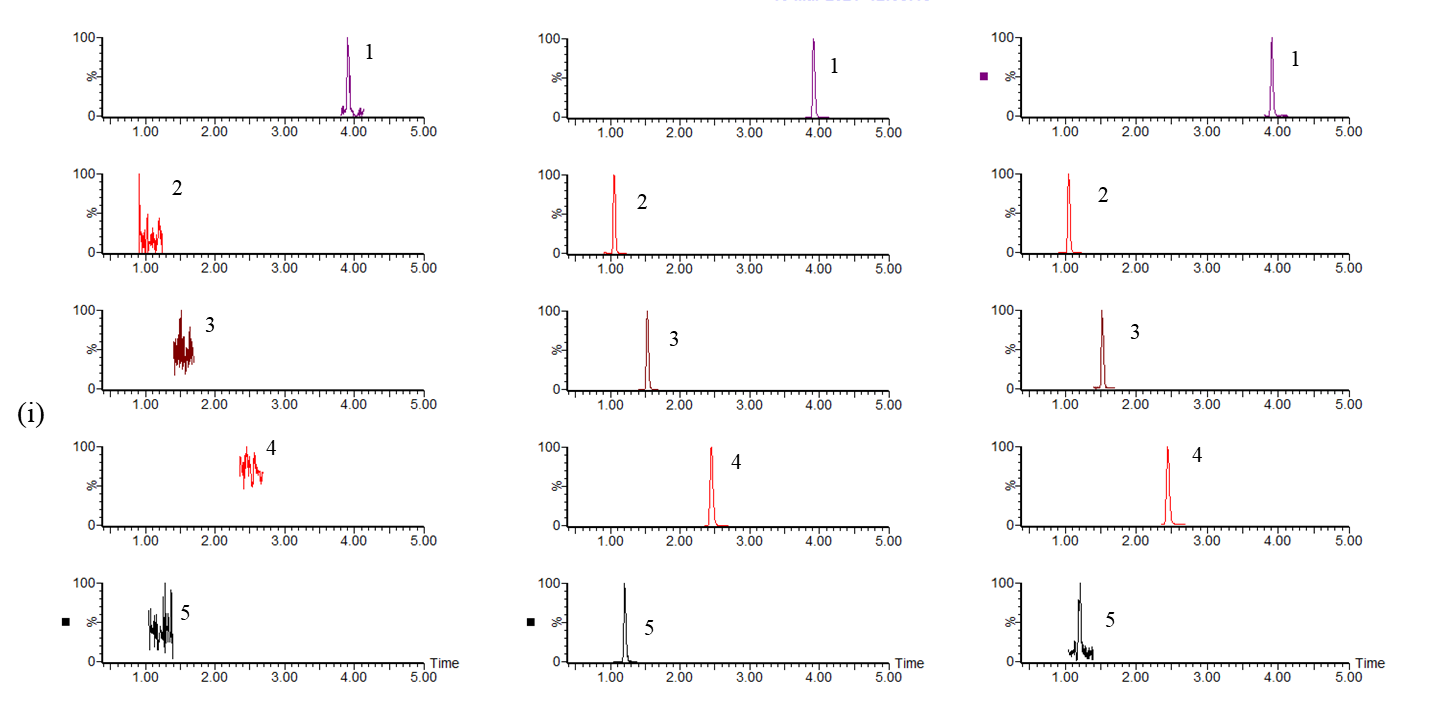


**Supplementary Figure S1.** Representative chromatograms. (A) blank plasma or tissue homogenate samples; (B) blank plasma or tissue homogenate samples spiked with the four components and IS; (C) plasma and tissue homogenate samples after oral administration of CA extract. (a) Plasma, (b) Heart, (c) Liver, (d) Kidney, (e) Lung, (f) Stomach, (g) Small intestine, (h) Spleen, (i) Brain. (1. qingyangshengenin; 2. puerarin; 3. deacylmetaplexigenin; 4. baishouwu benzophenone; 5.syringic acid)
